# Molecular Fingerprints for a Novel Enzyme Family in *Actinobacteria* with Glucosamine Kinase Activity

**DOI:** 10.1128/mBio.00239-19

**Published:** 2019-05-14

**Authors:** José A. Manso, Daniela Nunes-Costa, Sandra Macedo-Ribeiro, Nuno Empadinhas, Pedro José Barbosa Pereira

**Affiliations:** a IBMC-Instituto de Biologia Molecular e Celular, Universidade do Porto, Porto, Portugal; b i3S-Instituto de Investigação e Inovação em Saúde, Universidade do Porto, Porto, Portugal; c CNC-Center for Neuroscience and Cell Biology, University of Coimbra, Coimbra, Portugal; d PhD Program in Experimental Biology and Biomedicine (PDBEB), University of Coimbra, Coimbra, Portugal; e IIIUC-Institute for Interdisciplinary Research, University of Coimbra, Coimbra, Portugal; University of Illinois at Chicago

**Keywords:** *Streptacidiphilus jiangxiensis*, *Streptomycetaceae*, X-ray crystallography, antibiotic production, small-angle X-ray scattering

## Abstract

*Actinobacteria* have long been the main source of antibiotics, secondary metabolites with tightly controlled biosynthesis by environmental and physiological factors. Phosphorylation of exogenous glucosamine has been suggested as a mechanism for incorporation of this extracellular material into secondary metabolite biosynthesis, but experimental evidence of specific glucosamine kinases in *Actinobacteria* is lacking. Here, we present the molecular fingerprints for the identification of a unique family of actinobacterial glucosamine kinases. Structural and biochemical studies on a distinctive kinase from the soil bacterium Streptacidiphilus jiangxiensis unveiled its preference for glucosamine and provided structural evidence of a phosphoryl transfer to this substrate. Conservation of glucosamine-contacting residues across a large number of uncharacterized actinobacterial proteins unveiled a specific glucosamine binding sequence motif. This family of kinases and their genetic context may represent the missing link for the incorporation of environmental glucosamine into the antibiotic biosynthesis pathways in *Actinobacteria* and can be explored to enhance antibiotic production.

## Abstract (precis)

The discovery of novel enzymes involved in antibiotic biosynthesis pathways is currently a topic of utmost importance. The high levels of antibiotic resistance detected worldwide threaten our ability to combat infections and other 20th-century medical achievements, namely, organ transplantation or cancer chemotherapy. We have identified and characterized a unique family of enzymes capable of phosphorylating glucosamine to glucosamine-6-phosphate, a crucial molecule directly involved in the activation of antibiotic production pathways in *Actinobacteria*, nature’s main source of antimicrobials. The consensus sequence identified for these glucosamine kinases will help establish a molecular fingerprint to reveal yet-uncharacterized sequences in antibiotic producers, which should have an important impact in biotechnological and biomedical applications, including the enhancement and optimization of antibiotic production.

## INTRODUCTION

The worrying worldwide escalation of antimicrobial resistance is limiting the treatment options for numerous infectious diseases (1). Therefore, finding new strategies that lead to either novel effective antibiotics or enhancement of the production of the known drugs stands as a global challenge of utmost importance (2).

*Actinobacteria* have long been the largest natural source of antibiotics (3). Two-thirds of the currently known antibiotics are produced by organisms of the genus *Streptomyces*, the largest (>800 valid species) in the phylum *Actinobacteria* and a paradigm of secondary-metabolite-producing microorganisms (4). *Streptomyces* spp. belong to the *Streptomycetaceae* family, which so far includes only three additional genera, *Allostreptomyces*, *Kitasatospora*, and *Streptacidiphilus*, all very difficult to differentiate both by genotypic and by phenotypic characteristics (5, 6).

Postgenomic-era advances in the regulation of antibiotic production in *Actinobacteria* resulted from the identification of one of the most important regulators of *N*-acetylglucosamine (GlcNAc) uptake and metabolism and of degradation of chitin to GlcNAc, DasR from the GntR family (7–10). GlcNAc is an important antibiotic elicitor, participating in signaling pathways leading to secondary metabolite production in *Streptomyces* (11–13) and providing the glycosyl moieties of some antibiotics (14).

A central molecule of GlcNAc metabolism with a crucial role in cell wall synthesis, glycolysis, and nitrogen metabolism is glucosamine-6-phosphate (GlcN-6P) (15–18). Moreover and most importantly, GlcN-6P binds to DasR and modulates its DNA-binding activity, regulating the activation of antibiotic production in *Actinobacteria* (11–13, 19). The crystal structures of DasR and of its ortholog NagR from Bacillus subtilis in complex with GlcN-6P have been reported (20, 21). However, to date only two metabolic enzymes that lead to production of intracellular GlcN-6P in *Actinobacteria* have been described: *N*-acetylglucosamine-6-phosphate (GlcNAc-6P) deacetylase, NagA (EC 3.5.1.25), which deacetylates GlcNAc-6P formed by phosphorylation of acquired extracellular GlcNAc by a phosphotransferase system (22–25), and GlcN-6P synthase (EC 2.6.1.16), which produces GlcN-6P by transamination of the glycolytic intermediate fructose-6P (26, 27). Homologues of these two enzyme classes are present in the genomes of several *Streptomycetaceae*. On the other hand, it was recently demonstrated that the *csnR*-*K* operon, conserved in *Actinobacteria*, controls the uptake of chitosan-derived d-glucosamine (GlcN) oligosaccharides and, at a lower scale, of monomeric GlcN (28). Phosphorylation of exogenous GlcN to GlcN-6P could thus constitute an alternative mechanism for incorporation of extracellular material (chitin or chitosan) into the actinobacterial GlcN metabolism. Although GlcN kinases (EC 2.7.1.8) have been identified in the gammaproteobacterium Vibrio cholerae (29) and in the hyperthermophilic archaeon Thermococcus kodakarensis (30), and their existence in *Actinobacteria* has also been anticipated, allowing incorporation of extracellular material (chitin or chitosan) into the GlcN metabolism, experimental evidence for this activity in that medically important phylum is still missing. Here, we identify and characterize a unique GlcN kinase from the soil bacterium Streptacidiphilus jiangxiensis, closely related to *Streptomyces* spp., the classical antibiotic producers (31). This GlcN kinase is not a sequence homologue of the isofunctional enzymes from *Vibrio* or from archaea (29, 30). The crystal structure of the quaternary complex between this unusual kinase and GlcN, ADP, inorganic phosphate, and Mg^2+^ provides unparalleled structural evidence of a transition state of the phosphoryl-transfer mechanism in this unique family of GlcN kinases with a eukaryotic protein kinase fold. In addition, the molecular determinants for GlcN phosphorylation, with high evolutionary conservation in several representative families of the phylum *Actinobacteria*, have been determined, unveiling a sequence motif for the classification of a large number of uncharacterized or misannotated aminoglycoside phosphotransferases into this unique family of actinobacterial GlcN kinases.

## RESULTS

### A maltokinase paralogue associated with BGCs in *Actinobacteria*.

**FIG 1:**
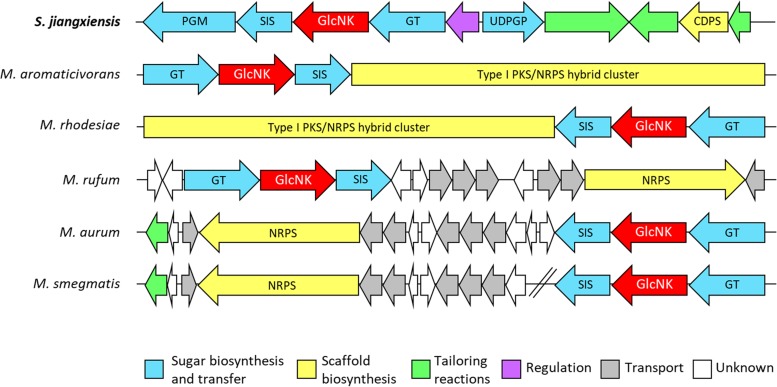
Genomic context of the Streptacidiphilus jiangxiensis maltokinase paralogue, SjGlcNK, and of its homologues in selected *Actinobacteria*. GlcNK is predicted to be within the borders of biosynthetic gene clusters in all represented species except for *Mycolicibacterium smegmatis*, which appears to have a cluster homologous to Mycolicibacterium rufum and Mycolicibacterium aurum but with the sugar-modifying genes, including GlcNK, located elsewhere on the chromosome. Figure drawn according to antiSMASH (50) results. PGM, putative phosphoglucomutase; SIS, putative phosphosugar isomerase; GlcNK, GlcN kinase; GT, putative glycosyltransferase; UDPGP, putative UTP–glucose-1-phosphate uridylyltransferase; CDPS, putative cyclodipeptide synthase; NRPS, putative nonribosomal peptide synthase; PKS, putative polyketide synthase.

In a few actinobacterial genomes, a gene with sequence homology to maltokinases (and annotated as such) was identified, in addition to the canonical maltokinase gene (32). In each species, the sequence identity between the canonical maltokinase and its paralogue was approximately 30%. Maltokinases catalyze the synthesis of maltose-1-phosphate, a key metabolite in α-glucan biosynthesis in actinomycetes (33). The presence of a putative second maltokinase gene in some *Actinobacteria* prompted a careful analysis of its genomic context, which revealed a tight association with two other uncharacterized genes, a putative sugar isomerase (SIS) of the AgaS superfamily (COG2222) and a putative glycosyltransferase (GT) with homology to members of the GT1 family (Fig. 1). In Streptacidiphilus jiangxiensis, two other genes with possible sugar-modifying activities are present, annotated as putative phosphoglucomutase (PGM) and UTP–glucose-1-phosphate uridylyltransferase (UDPGP), thus completing a potential pathway for sugar activation and transfer. Furthermore, in several *Actinobacteria*, this operon was predicted to be included in larger biosynthetic gene clusters (BGCs), suggesting a role in the production of complex secondary metabolites (Fig. 1). Since the Streptacidiphilus jiangxiensis homologue was part of an apparently more complete operon and given the phylogenetic relationship of this organism with antibiotic-producing *Streptomyces*, this gene was selected for further investigation.

### The *S. jiangxiensis* maltokinase paralogue encodes an enzyme with GlcN kinase activity.

**FIG 2:**
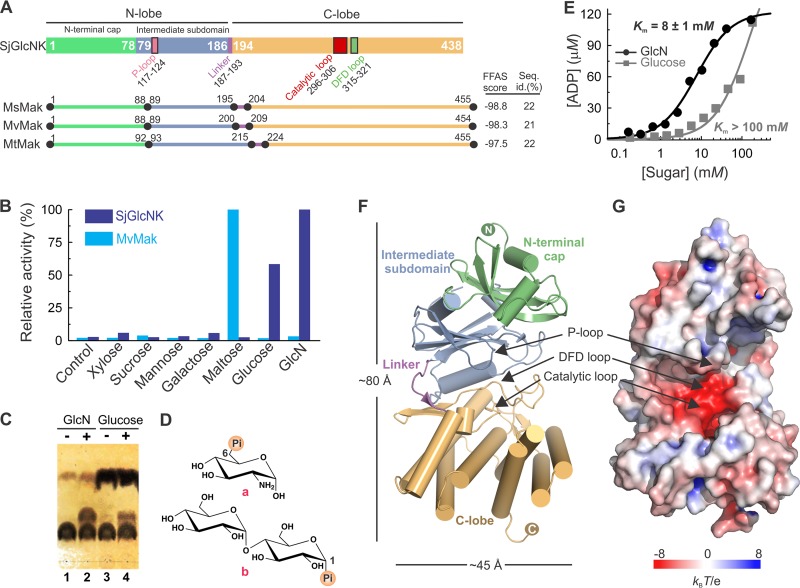
Biochemical characterization and overall structure of SjGlcNK. (A) Structural organization of SjGlcNK (colored rectangles) and of the mycobacterial maltokinases MsMak (PDB entry 5JY7), MvMak (PDB entry 4U94 [35]), and MtMak (PDB entry 4O7O [36]) (colored lines), indicating the relative arrangement of the N-terminal cap (light green) and intermediate subdomains (blue), linker (magenta), and C-lobe (orange). The canonical structural motifs in SjGlcNK—P-loop (pink), catalytic loop (red), and DFD loop (green)—are also indicated. The FFAS score (34) and the percentage of amino acid sequence identity between SjGlcNK and each of the mycobacterial maltokinases are given to the right. (B) Relative kinase activity of SjGlcNK (dark blue) and MvMak (cyan) toward different potential phosphate acceptors (xylose, sucrose, mannose, galactose, maltose, glucose, and GlcN). The reaction mix ([enzyme] = 2 μM, [sugar] = 5 mM, [ATP] = 1 mM) was incubated at room temperature for 5 min, and activity was quantified with the ADP-Glo kinase assay. The results were normalized against the luminescence signal for the reactions with maltose (MvMak) or GlcN (SjGlcNK). While MvMak uses exclusively maltose as the substrate acceptor, SjGlcNK phosphorylates glucose and GlcN, with preference for the latter. As a control, the activity in the absence of phosphate-accepting sugars is shown. (C) Thin-layer chromatography analysis of SjGlcNK-catalyzed phosphorylation of GlcN and glucose using ATP as phosphate donor. Lanes 1 and 3, negative controls without SjGlcNK; lane 2, reaction with GlcN; lane 4, reaction with glucose. (D) Chemical structures of the reaction products of SjGlcNK and MvMak, GlcN-6P (a) and maltose-1-phosphate (b), respectively. (E) Kinetics of SjGlcNK activity toward glucose and GlcN. The experimental data were fitted to the Michaelis-Menten equation. The measured *K_m_* for GlcN (black) is more than 10-fold lower than that for glucose (gray), in agreement with the observed preference for this substrate. (F) Cartoon representation of the three-dimensional structure of SjGlcNK. The N-terminal cap, intermediate subdomain, linker, and C-lobe are labeled and colored as in panel A. Arrows indicate the positions of the P-loop, DFD loop, and catalytic loop. (G) Solid surface representation of SjGlcNK colored according to the electrostatic potential contoured from −8 (red) to 8 (blue) *k_B_T*e^−1^ (*k_B_*, Boltzmann’s constant; *T*, temperature [K]; e, charge of an electron) highlighting the acidic nature of the active site located in the groove between the intermediate subdomain and the C-lobe.

In order to gain more insight into the potential function of the *S. jiangxiensis* maltokinase paralogue, here SjGlcNK, a sequence profile-based search for homologues was performed using the Fold and Function Assignment System (FFAS03) (34). As expected, three significant hits were identified corresponding to the maltokinases from Mycolicibacterium smegmatis (MsMak; PDB entry 5JY7, FFAS score = −98.8),Mycolicibacterium vanbaalenii (MvMak; PDB entry 4U94 [35], FFAS score = −98.3), and Mycobacterium tuberculosis (MtMak; PDB entry 4O7O [36], FFAS score = −97.5). FFAS03 scores below −9.5 correspond to high-confidence predictions with less than 3% false positives. The mycobacterial homologues identified display 21% to 22% amino acid sequence identity to SjGlcNK, with many of the residues of the canonical structural motifs associated with nucleotide binding and enzymatic activity in the maltokinases being highly conserved in SjGlcNK (see Fig. S1 in the supplemental material). Despite a slightly shorter sequence, the important catalytic motifs P-loop (^117^VDQTNESV^124^) (35) and the ^130^AVVKW^134^ region containing the conserved phosphate-binding lysine residue, as well as the catalytic (^296^DVHGDFHVGQI^306^) (37) and ^317^DFD^319^ (38) loops could be identified in SjGlcNK (Fig. S1; Fig. 2A). Interestingly, SjGlcNK was unable to phosphorylate maltose *in vitro* (Fig. 2B), indicating an incorrect annotation of its function. In contrast, from 20 sugars and 4 aminoglycoside antibiotics tested, SjGlcNK only phosphorylated specifically GlcN and displayed vestigial activity with glucose using ATP as a phosphate donor (Fig. 2C). GlcN was modified at position 6, yielding GlcN-6P, as determined by nuclear magnetic resonance (NMR) (Fig. 2D; Fig. S2). Determination of the Michaelis constants (*K_m_*) for glucose and GlcN suggested that SjGlcNK is better described as a GlcN kinase (EC 2.7.1.8) with a strong preference for using GlcN (*K_m_* = 8 ± 1 mM) over glucose (*K_m_* > 100 mM) as the substrate (Fig. 2E).

10.1128/mBio.00239-19.1 FIG S1 Multiple amino acid sequence alignment of SjGlcNK with homologous mycobacterial maltokinases. The amino acid sequence of SjGlcNK from Streptacidiphilus jiangxiensis (UniProtKB entry A0A1H7TQR5) was aligned with those of MvMak from *Mycolicibacterium vanbaalenii* (UniProtKB entry A1TH50), MtMak from Mycobacterium tuberculosis (UniProtKB entry O07177), and MsMak from *Mycolicibacterium smegmatis* (UniProtKB entry A0R6D9). Strictly conserved alignment positions are shown in inverted type on a red background. Secondary structure elements for SjGlcNK are represented above the alignment. The catalytic and DFD loops, the P-loop, and the DVV and AVVKW motifs are labeled in red. The residues that participate in GlcN and maltose binding are indicated by green and orange hexagons, respectively. Download FIG S1, PDF file, 2.2 MB. Copyright © 2019 Manso et al. 2019 Manso et al. This content is distributed under the terms of the Creative Commons Attribution 4.0 International license.

10.1128/mBio.00239-19.2 FIG S2 ^1^H-NMR spectra of the enzymatic product of SjGlcNK. (A) Sample from enzymatic reaction mixture purified by thin-layer chromatography. (B) GlcN-6P standard (Sigma-Aldrich) in D_2_O. (C) Sample from spectrum A spiked with an aliquot of standard B. The alignment between the signals in samples A and B is perfect. The spiking of sample A with the standard revealed no new signals and a slight increase of those already present in the sample, thus confirming the identity of the sample compound as GlcN-6P. Download FIG S2, PDF file, 0.1 MB. Copyright © 2019 Manso et al. 2019 Manso et al. This content is distributed under the terms of the Creative Commons Attribution 4.0 International license.

### SjGlcNK displays a fold similar to mycobacterial maltokinases.

The crystal structure of SjGlcNK was solved at 1.98-Å resolution (Table 1) and reveals a typical eukaryotic protein kinase fold (37, 39), consisting of an N-lobe (formed by an N-terminal cap and an intermediate subdomain) and a C-lobe separated by a linker, with a relative arrangement that results in an overall bean shape (Fig. 2F). The important conserved catalytic motifs, the DFD loop (38) and the catalytic loop (37), are located in an acidic groove between the two lobes, whereas the P-loop (35) is in the vicinity of this patch (Fig. 2G). Structural differences between SjGlcNK and mycobacterial maltokinases are mainly found in the N-terminal cap subdomain. The SjGlcNK N-terminal cap is composed of three long antiparallel β-strands (βA-βB-βC) forming a curved β-sheet enclosing α-helices α1 and α2 and by an additional β-strand (βD) perpendicular to strand βB on its concave surface (Fig. S3A). In contrast, the N-terminal cap subdomains of mycobacterial maltokinases are 10 to 14 residues longer than that of SjGlcNK (Fig. 2A) and display additional secondary structural elements (e.g., β4* and αB*), absent in SjGlcNK (Fig. S3A). These differences could be explained in terms of the different genetic context for the two enzymes, since the unique N-terminal cap of mycobacterial maltokinases is in some cases fused to the C terminus of trehalose synthase (35, 40). The intermediate subdomain of SjGlcNK, which is composed of a six-stranded β-sheet (βF-βG-βH-βJ-βI-βE) flanked by two α-helical segments (α3 and α4), is almost identical to that of mycobacterial maltokinases, with a root mean square deviation (RMSD) of 1.7 Å for 80 aligned Cα atoms (Fig. S3B). A seven-residue linker (residues 187 to 193) connects the intermediate subdomain to the C-terminal lobe (residues 194 to 438), which is composed of two central 4-helical bundles (α5-α6-α10-α11 and α7-α8-α12-α13) and a small two-stranded β-sheet (βK-βL), with an additional helix α9, also present in MtMak, preceding βK and downstream of the catalytic loop. The C-terminal lobe is very similar to the corresponding domain of maltokinases with an RMSD of 1.2 Å upon superposition of 140 Cα atoms (Fig. S3C).

**TABLE 1:** Crystallographic data collection and refinement statistics

	Apo SjGlcNK (crystal form A, rendering conformations I and VI)^*a*^	Apo SjGlcNK (crystal form B, rendering conformations II, III, IV, and V)^*a*^	Complex of SjGlcNK with ADP, P_i_, and GlcN (crystal form A, rendering conformations I and VI)^*a*^
Data collection			
X-ray beamline	BL13-XALOC (ALBA)	ID30A-3 (ESRF)	ID30A-3 (ESRF)
Space group	*P*2_1_	*P*2_1_	*P*2_1_
Cell dimension			
*a* (Å)	59.8	58.2	58.8
*b* (Å)	96.1	111.2	97.4
*c* (Å)	80.3	150.6	80.2
β (°)	106.7	93.2	107.4
Wavelength (Å)	1.0332	0.9677	0.9677
Resolution range (Å)	40.7–1.98 (2.01–1.98)	47.3–2.69 (2.74–2.69)	60.2–2.15 (2.19–2.15)
Total/unique reflections	207,505/59,488 (10,538/2,936)	366,841/52,817 (19,263/2,664)	325,525/46,229 (17,142/2,340)
Avg multiplicity	3.5 (3.6)	6.9 (7.2)	7.0 (7.3)
Completeness (%)	98.1 (97.3)	98.9 (100.0)	98.3 (99.2)
*R*_meas_^*b*^ (%)	6.7 (106.7)	22.5 (126.2)	12.2 (166.0)
CC_1/2_ (%)	99.9 (58.4)	98.6 (59.0)	99.8 (55.9)
Mean *I*/σ*I*	13.2 (1.3)	9.6 (2.2)	11.8 (1.3)
Refinement			
Resolution range (Å)	40.7–1.98	47.3–2.69	39.9–2.15
Unique reflections, work/free	59,479/2,962	52,795/2,646	46,219/2,320
R work (%)	17.6	20.6	20.7
R free^*c*^ (%)	21.4	24.2	24.3
No. of:			
Amino acid residues	833	1,639	817
Water molecules	464	256	243
Mg^2+^	7	3	2
Cl^−^	4	4	2
Bis-Tris propane	2		1
PEG	3		3
ADP			1
Phosphate ion			1
GlcN			1
Avg B value (Å^2^)			
Wilson plot	34.6	42.0	41.3
Protein	39.3 (I)/54.2 (VI)	44.8 (III)/50.0 (II)/46.6 (IV)/42.7 (V)	45.7 (I)/62.9 (VI)
Solvent	49.0	38.6	50.1
Mg^2+^	53.5	42.9	52.6
Cl^−^	33.7	33.4	38.3
Bis-Tris propane	96.7		61.8
PEG	88.8		72.5
ADP			79.8
Phosphate ion			66.8
GlcN			73.7
RMSD bond length (Å)	0.006	0.002	0.002
RMSD bond angle (°)	0.672	0.450	0.498
Ramachandran plot,^*d*^ no. of residues (%) in:			
Favored regions	801 (97.7)	1,543 (96.5)	776 (97.4)
Additionally allowed	19 (2.3)	55 (3.4)	21 (2.6)
Outliers	0	1 (0.1)	0
PDB code	6HWJ	6HWK	6HWL

a Numbers in parentheses correspond to the outermost resolution shell.

b *R*_meas_ is the multiplicity-independent *R* factor (54).

c Calculated using 5% of reflections that were not included in the refinement.

d As defined in MOLPROBITY (55).

10.1128/mBio.00239-19.3 FIG S3 Overall structure of SjGlcNK and structural comparison with MvMak. (A) Structural superposition of SjGlcNK (colored as in Fig. 2F) and MvMak (gray; PDB entry 4U94 [J. Fraga, A. Maranha, V. Mendes, P. J. B. Pereira, N. Empadinhas, et al., Sci Rep 5:8026, 2015, https://doi.org/10.1038/srep08026]) N-terminal caps (two views rotated by 180° around *y*), (B) intermediate subdomains, and (C) C-lobes (two views rotated by 90° around *y*). Secondary structure elements are labeled. Download FIG S3, PDF file, 2.5 MB. Copyright © 2019 Manso et al. 2019 Manso et al. This content is distributed under the terms of the Creative Commons Attribution 4.0 International license.

### Conformational flexibility regulates the catalytic activity of SjGlcNK.

**FIG 3:**
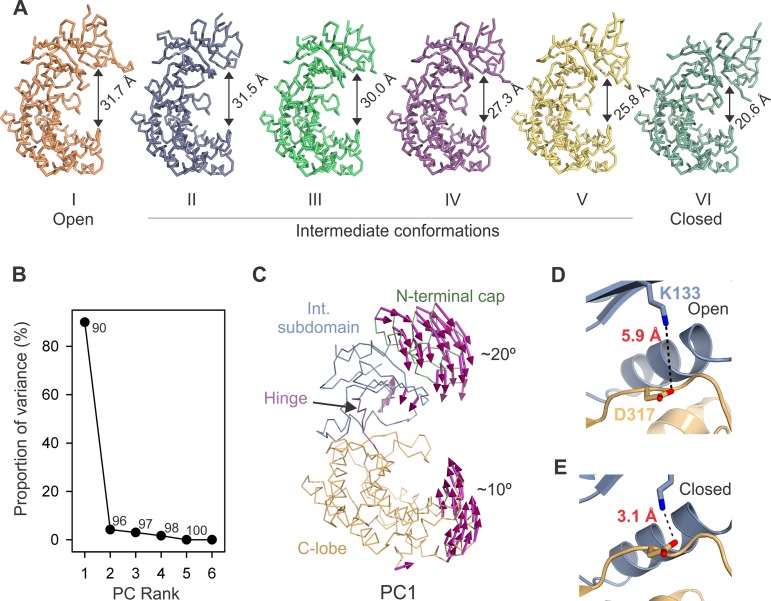
SjGlcNK is highly flexible. (A) Ribbon representation of six crystallographic models of SjGlcNK, highlighting their different conformational states. Differences in the relative position of the two lobes (the measured distance between the Cα atoms of A15 and H416 is indicated for each state) indicate conformational flexibility. The structures are labeled with Roman numerals and ordered from left to right, according to decreasing interlobe distance. (B) Principal-component (PC) analysis of subdomain-wide conformational differences between the six crystallographic models. The plot indicates the percentage of the total variance captured by each eigenvector, with numbers indicating the cumulative variance accounted for all preceding eigenvectors. (C) Porcupine illustration of the PC1 that relates the six individual SjGlcNK structures. Each subdomain is labeled and colored as in Fig. 2F. (D and E) Closeup view of part of the active site of SjGlcNK in the open (D) and closed (E) conformation. The distance between the catalytic K133 and D317 is indicated.

SjGlcNK was crystallized in two different monoclinic (*P*2_1_) crystal forms, A and B, which differ considerably in the dimensions of the respective unit cells (Table 1). In consequence, there are two SjGlcNK molecules in the asymmetric unit (AU) of crystal form A, while four monomers are found in the larger AU of crystal form B (Fig. S4A). The individual structures of the N-terminal cap, the intermediate subdomain, and the C-lobe are highly conserved in the six protomers, across the two crystal forms. In accordance, pairwise superposition of the six structures results in an RMSD of 0.26 to 0.51 Å when the N-terminal cap is aligned and of 0.30 to 0.50 Å or 0.29 to 0.43 Å when aligning the intermediate subdomain or the C-lobe, respectively. In contrast to the structural conservation of the individual subdomains, the relative positions of the N- and C-lobes are strikingly different in the six molecules (Fig. S4B). In result, the distance between equivalent atoms in the N- and C-lobe varies from 31.7 Å in the open to 20.6 Å in the closed conformation (Fig. 3A), and superposition of the C-lobe (residues 194 to 420) of the six different conformational states results in RMSD values ranging from 0.6 to 5.6 Å for the whole molecule. Principal-component (PC) analysis revealed that the six conformers of SjGlcNK are related by an opening-closure movement (PC1), i.e., a hinge-bending motion of the N- and C-lobes of ∼30° amplitude (Fig. 3B and C). In this movement, the linker connecting the two lobes (residues 187 to 191) acts as a hinge and the catalytic lysine, K133, which sits far from the active site in the open state I (Fig. 3A and D), approaches and establishes contacts with the catalytic D317 in the closed state VI (Fig. 3A and E), likely corresponding to the active conformation of the enzyme.

10.1128/mBio.00239-19.4 FIG S4 The two crystallographic forms, A and B, of SjGlcNK comprise six conformational states of the enzyme. (A) Cartoon representation of two (left, crystal form A) and four (right, crystal form B) molecules found in the asymmetric units of the two SjGlcNK crystal forms. The individual structures are labeled with Roman numerals. (B) Superposition of the Cα traces of the six SjGlcNK monomers (colored as in panel A), by alignment of the C-lobe subdomains. The orientations of the N-lobe in the open and closed conformations are related by a rotation of 23.8° around a hinge axis (dashed line) as determined by DynDom (S. Hayward and H. J. Berendsen, Proteins, 30:144–154, 1998, https://doi.org/10.1002/(SICI)1097-0134(19980201)30:2%3C144::AID-PROT4%3E3.0.CO%3B2-N). Download FIG S4, PDF file, 1.0 MB. Copyright © 2019 Manso et al. 2019 Manso et al. This content is distributed under the terms of the Creative Commons Attribution 4.0 International license.

The quaternary structure and the conformational state of SjGlcNK in solution were elucidated using a combination of small-angle X-ray scattering (SAXS) and X-ray crystallography. In solution, SjGlcNK is a monomer (Table S1), adopting an open conformation in the absence of potential substrates (Fig. S5A). By itself, neither GlcN nor glucose induces apparent conformational changes in SjGlcNK. In contrast, the presence of ATP results in a marked increase in the contribution of closed conformations to the overall scattering. This, together with a decrease in the Guinier radius of gyration (*R_g_*; Fig. S5B) suggests that upon ATP binding SjGlcNK assumes a more compact and closed conformation. The contribution of a closed conformation to the overall scattering is even slightly larger when SjGlcNK is simultaneously in the presence of ATP and a sugar substrate (Fig. S5), suggesting a concerted mechanism where the sugar substrate is phosphorylated in the closed conformation of SjGlcNK. In summary, ATP induces the closure of SjGlcNK, and the crystallographic structures reported represent six different conformational snapshots of the enzyme, arising from the molecular flexibility required to carry out its catalytic cycle.

10.1128/mBio.00239-19.5 FIG S5 Ligand-induced conformational transformation of SjGlcNK in solution probed by SAXS. (A) SAXS profiles extrapolated to infinite dilution of SjGlcNK (a) in absence of substrates (red) and in presence of (b) 200 mM GlcN (blue), (c) 50 mM glucose (violet), (d) 1 mM ATP (orange), (e) 200 mM GlcN and 1 mM ATP (green), and (f) 50 mM glucose and 1 mM ATP (yellow). The curves are offset on the log scale. The scattering calculated for a combination of open and closed conformations (ratio given above the curves) is shown as dashed lines. (B) Guinier plots of the scattering data shown in panel A. *R_g_* values (±SD) are indicated for each experiment. (C) Plot of Guinier *R_g_* at several concentrations, for the same combinations of protein and ligand(s) as in panel A. Download FIG S5, PDF file, 0.3 MB. Copyright © 2019 Manso et al. 2019 Manso et al. This content is distributed under the terms of the Creative Commons Attribution 4.0 International license.

10.1128/mBio.00239-19.8 TABLE S1 Small-angle X-ray scattering results for SjGlcNK with and without ligands. Download Table S1, DOCX file, 0.1 MB. Copyright © 2019 Manso et al. 2019 Manso et al. This content is distributed under the terms of the Creative Commons Attribution 4.0 International license.

### Caught in the act: crystal structure of a productive complex of SjGlcNK.

**FIG 4:**
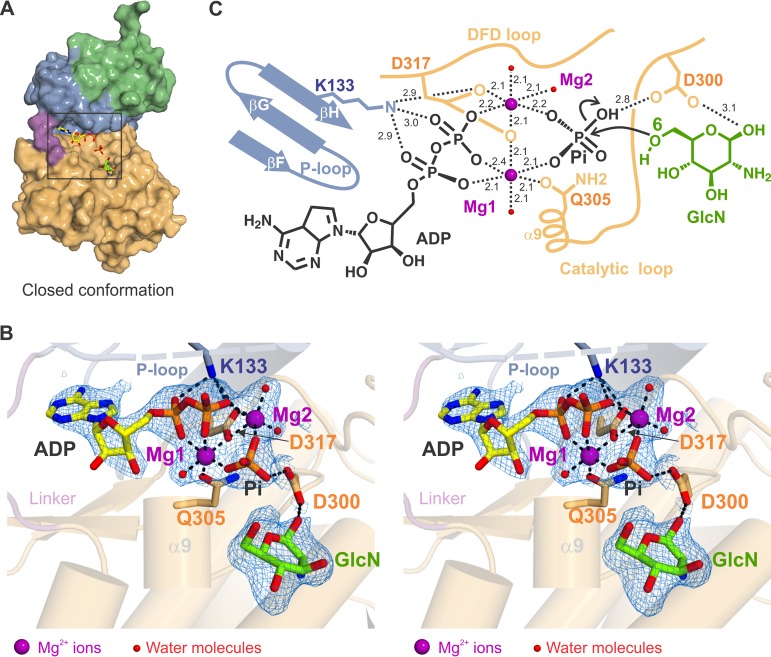
The crystal structure of a productive complex of SjGlcNK unveils the mechanism of phosphate transfer. (A) Surface representation of the SjGlcNK-GlcN-ADP-P_i_ quaternary complex with subdomains colored as in Fig. 2F. A square highlights the position of the substrates (stick representation) in the active site. (B) Closeup stereo view of the enzyme active site in the quaternary complex. The electron density map (2*mF*o-*DF*c contoured at 1.0 σ) around ADP, P_i_, GlcN, Mg^2+^ ions and Mg^2+^-coordinating water molecules is shown as a blue mesh. The substrates are shown as sticks with oxygen atoms red, nitrogen blue, phosphorus orange, and carbon yellow (ADP) or green (GlcN). Metal coordination and hydrogen bonds are indicated by black dashed lines. The protein (intermediate subdomain, linker, and C-lobe containing the catalytic helix α9) is depicted in cartoon representation and color coded as in Fig. 2F. Catalytic residues K133, D300, Q305, and D317 are represented as sticks (carbon atoms colored according to subdomain color scheme). (C) Schematic representation of the transition state of GlcN phosphorylation by SjGlcNK. Magnesium ion coordination and polar interactions are shown as black dashed lines, labeled with the respective distances in Å. Magnesium ions and water molecules are shown as magenta and red spheres, respectively.

In crystals obtained by cocrystallization with a molar excess of ATP and GlcN (Table 1), the substrates could be easily located in the electron density map, at the active site of the enzyme (Fig. S6A). Interestingly, this structure represents a snapshot of the phosphoryl transfer reaction in which ATP is hydrolyzed into ADP and inorganic phosphate (P_i_), with the two hydrolysis products clearly identifiable in the electron density map. The ADP moiety is located in a cleft between the two lobes of SjGlcNK, which adopts a closed conformation (Fig. 4A), while both P_i_ and GlcN are found at the C-lobe. Binding of ADP to SjGlcNK is similar to the interaction between the nucleotide and mycobacterial maltokinases (35). The adenine group binds to the hinge region, cross-linking the two lobes of the enzyme. In particular, the adenine establishes hydrophobic contacts with Y187 and L188 at the hinge, with V131, P164, and V185 at the intermediate subdomain, and with L307 and V316 at the C-lobe. In addition, both the adenine and ribose moieties establish polar contacts with the main chain of A186 and L188 and with the side chain of D193, residues located at the hinge. A major consequence of this extensive network of contacts is the observed ATP-induced closure of SjGlcNK (see above).

10.1128/mBio.00239-19.6 FIG S6 Crystal structure of the complex between SjGlcNK, GlcN and ATP. (A) The structure of the complex reveals the transition state of the phosphoryl transfer reaction of ATP to GlcN. Although a residual, noninterpretable positive electron density was observed near the active site of the closed state (VI in Fig. 3A) of apo-SjGlcNK (crystal form A), in crystals obtained by cocrystallization with a molar excess of ATP and GlcN (Table 1) the substrates could be easily located in the electron density map. The structure of SjGlcNK (closed conformation) is shown in ribbon representation colored as in Fig. 2F. The Polder *mF*_o_-*DF*_c_ map (D. Liebschner, P. V. Afonine, N. W. Moriarty, B. K. Poon, O. V. Sobolev, et al., Acta Crystallogr D Biol Crystallogr 73:148–157, 2017, https://doi.org/10.1107/S2059798316018210) (contoured at 4σ) around the ADP, inorganic phosphate (P_i_), magnesium ions (Mg1 and Mg2), and GlcN is shown as a green mesh. (B) Ligand binding induces the reorientation of the side chain of D317. Magnesium binding sites are shown for the nucleotide-free SjGlcNK structure (left) and for the phosphorylation transition state (right) found in the GlcN-ADP-P_i_ quaternary complex. Download FIG S6, PDF file, 1.5 MB. Copyright © 2019 Manso et al. 2019 Manso et al. This content is distributed under the terms of the Creative Commons Attribution 4.0 International license.

This crystal structure represents a transient state of GlcN phosphorylation, in which all participating components—ADP, P_i_, two magnesium ion cofactors (Mg1 and Mg2), and GlcN—were captured midreaction, providing a detailed mechanistic insight into phosphoryl transfer (Fig. 4B and C). In this transition state, the energetically stabilized octahedral coordination of the two magnesium ions drives the breakdown of ATP into ADP and P_i_, similar to what is observed for the cAMP-dependent protein kinase (PKA) (41–44). The catalytic residues D317 (in the DFD loop) and Q305 (in the catalytic loop) coordinate the two magnesium ions, stabilizing this transition state. In particular, D317 plays an important role for positioning the Mg1 and Mg2 cations. Curiously, the conformation of the side chain of D317 changes between the nucleotide-free (where it is coordinating the single and more tightly bound Mg1) and the quaternary complex structure (where it coordinates the two magnesium ions; Fig. S6B). The absence of the Mg2 ion in the nucleotide-free structures is not unexpected, since the loss of Mg2 is likely the rate-limiting step of ADP release, as reported in the PKA phosphoryl transfer mechanism (44).

Although K133 establishes hydrogen bonds with the α and β phosphate groups of ADP in the quaternary complex, this interaction is not key for anchoring the nucleotide to the active site (45). As observed in the SAXS experiments, ATP binding induces more compact and closed conformations of SjGlcNK, bringing K133 closer to the active site (Fig. 3D and E). The positive charge of K133 increases the electrophilicity of the γ-phosphate group of ATP, facilitating its hydrolysis (46). In this complex, the leaving phosphate is stabilized by D300 and the two magnesium ions, being perfectly oriented for transfer to position 6 of the bound sugar substrate, GlcN. This unique crystal structure provides experimental evidence of a phosphoryl transfer to GlcN with unprecedented atomic detail on the transition state of the general mechanism of phosphorylation by eukaryotic protein kinase-like kinases.

### The molecular determinants for substrate specificity in SjGlcNK are highly conserved in *Actinobacteria*.

**FIG 5:**
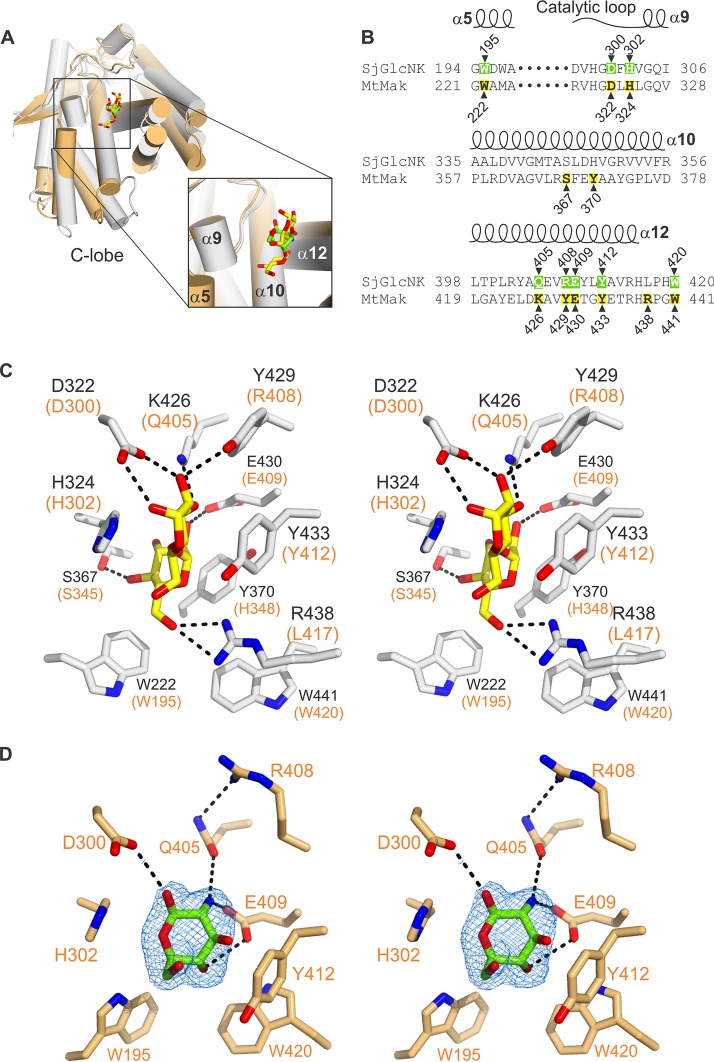
Molecular determinants for substrate specificity in SjGlcNK. (A) Cartoon representation of the superposed C-lobes of MtMak (gray, PDB code 4O7P) and SjGlcNK (orange) in complex with maltose (carbon atoms yellow) and GlcN (carbon atoms green), respectively. The sugar-binding site is highlighted by a square, shown in closeup to the right (key secondary structural elements for the interaction labeled). (B) Amino acid sequence alignment of the sugar-binding site of SjGlcNK with that of the mycobacterial maltokinase MtMak (UniProtKB entry O07177). The amino acids interacting with GlcN and maltose are highlighted in green and yellow, respectively. Secondary structure elements for SjGlcNK are represented above the alignment. (C) Closeup stereo view of the maltose binding site of MtMak (36). The amino acids involved in maltose binding and maltose are shown as sticks with oxygen atoms red, nitrogen blue, and carbon gray (protein) or yellow (maltose). Residues interacting with maltose are labeled. The equivalent residues in SjGlcNK are indicated in orange parentheses. (D) Closeup stereo view of the GlcN binding site of SjGlcNK. The amino acids involved in GlcN binding and GlcN are shown as sticks with oxygen atoms red, nitrogen blue, and carbon light orange (protein) or green (GlcN). Residues interacting with GlcN are labeled. The electron density map (2*mF*o-*DF*c contoured at 1.0 σ) around the bound GlcN molecule is shown as a blue mesh. Dashed black lines represent polar contacts.

**FIG 6:**
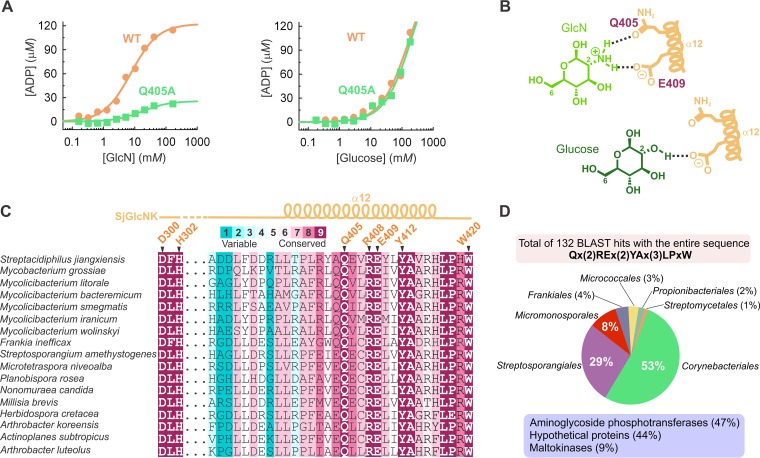
Unveiling the evolutionarily conserved residues for GlcN phosphorylation in *Actinobacteria*. (A) Effect of the structure-based amino acid replacement Q405A on the activity of SjGlcNK toward GlcN (left) and glucose (right). The replacement of Q405 by an alanine resulted in a marked decrease of the GlcN-phosphorylating activity of the enzyme, while no effect was observed for glucose, suggesting that Q405 is involved only in GlcN binding. Data were fitted to the Michaelis-Menten equation. (B) Schematic representation of the role of Q405 and E409 in GlcN and glucose binding. Both Q405 and E409 are ideally positioned to establish hydrogen bonds with the amino group of GlcN, with Q405 being key for GlcN specificity. A binding mode similar to GlcN was assumed for glucose. In contrast to GlcN, glucose establishes no interaction with Q405. (C) Multiple amino acid sequence alignment of regions 300 to 302 and 388 to 420 of SjGlcNK with the sequences of proposed homologous GlcN kinases from different representative species of *Actinobacteria*. Positions are colored according to evolutionary conservation, calculated from a multiple alignment of 89 unique sequences. The GlcN-contacting residues D300, H302, Q405, R408, E409, Y412, and W420 are highlighted and display strict conservation in these species of *Actinobacteria*. Secondary structure elements for SjGlcNK are represented above the alignment. (D) Pie chart indicating the distribution of the 132 hits returned by BLAST when querying the UniProt database with the consensus sequence across the different orders of the phylum *Actinobacteria*. The assigned functions of the hits are given in the mauve box.

The functional difference between SjGlcNK and mycobacterial maltokinases, despite their overall structural similarity, is an interesting riddle. Structural superposition of the C-lobes of MtMak and SjGlcNK in complex with maltose and GlcN, respectively, reveals that the sugar binding sites are located in the same position for the two enzymes, bordered by the catalytic loop and helices α5, α10, and α12 (Fig. 5A). Six conserved residues—W195, D300, H302, E409, Y412, and W420 (W222, D322, H324, E430, Y433, and W441 in MtMak)—mediate binding of the two sugars (Fig. 5B). Interestingly, Y370 and R438, which interact with the second glucose moiety of maltose in MtMak, are replaced by H348 and L417 in SjGlcNK, which are unable to establish equivalent contacts (Fig. 5C). In addition, region ^405^QEVR^408^ in helix α12 (^426^KAVY^429^ in MtMak) also plays a part in determining substrate specificity. Noteworthy is the role of Q405, stabilized by polar contacts with R408, in providing specificity to this family of GlcN kinases. The only difference between GlcN and glucose resides at position 2, where the amino group of GlcN is replaced by a hydroxyl. In SjGlcNK, the amino group of GlcN is within hydrogen-bonding distance of the side chains of Q405 (K426 in MtMak) and E409 (E430 in MtMak; Fig. 5D). When Q405 was replaced by an alanine residue in SjGlcNK, its activity remained unchanged toward glucose but was largely reduced with GlcN (Fig. 6A). The activity data for SjGlcNK variant Q405A suggest that Q405 is involved only in binding GlcN but not glucose (Fig. 6B), therefore conferring specificity for the former. Additionally, the amino group of GlcN is mildly basic (pK_a_ 7.58 [47]) and is likely protonated in the overall acidic environment of the active site (Fig. 2G). The resulting opposite charges of the substrate and the active site E409 arguably result in an additional electrostatic component to the specific recognition of GlcN by SjGlcNK (Fig. 6B). Importantly, Q405 and other GlcN-contacting amino acids (D300, H302, R408, E409, Y412, and W420) are highly and exclusively conserved in a large number of uncharacterized aminoglycoside phosphotransferases or putative maltokinases (with lower prediction score) belonging to several representative orders of the phylum *Actinobacteria* (Fig. 6C). In addition, residues A413, L417, and P418 are also highly conserved. Hence, we propose the consensus sequence “Q-x(2)-RE-x(2)-YA-x(3)-LP-x-W” as a signature for the identification of GlcN kinases, namely, those still unclassified in members of the phylum *Actinobacteria*, such as the one described here. Indeed, a BLAST search for this consensus sequence in the nonredundant protein sequence database identified 132 sequences, annotated as putative maltokinases, hypothetical proteins, or aminoglycoside phosphotransferases of unknown function, all of them from organisms belonging to the phylum *Actinobacteria* (Fig. 6D). Interestingly, one of these sequences corresponds to the maltokinase paralogue from M. smegmatis (UniProtKB entry A0A0D6IZ29), MsGlcNK. The substrate specificity of MsGlcNK was also investigated, revealing that it phosphorylates GlcN with a *K_m_* very similar to that of SjGlcNK, being inactive toward maltose (Fig. S7). Thus, the consensus sequence now identified for this unique family of proteins and experimentally validated for one of the hits (MsGlcNK) will certainly help to correctly identify and annotate a large number of actinobacterial enzymes, currently with no assigned function or incorrectly annotated as maltokinases.

10.1128/mBio.00239-19.7 FIG S7 The putative maltokinase from M. smegmatis, MsGlcNK, phosphorylates GlcN. Similarly to SjGlcNK, MsGlcNK displays preference for GlcN over maltose as the substrate. The enzymatic activity was measured as ADP release from ATP using the ADP-Glo kinase assay kit (Promega) (H. Zegzouti, M. Zdanovskaia, K. Hsiao, and S. A. Goueli, Assay Drug Dev Technol, 7:560–572, 2009, https://doi.org/10.1089/adt.2009.0222). Kinase reactions were performed in 100 mM Tris-HCl pH 7.5, 20 mM MgCl_2_, 0.1 mg ml^−1^ BSA with [MsGlcNK] = 1 μM, [ATP] = 2.5 mM, and various concentrations of GlcN (red dots) and maltose (black dots), upon incubation at room temperature for 5 min. Fitting the data to the Michaelis-Menten equation (red line) resulted in a *K_m_* of 14 ± 2 mM, very similar to that of SjGlcNK (*K_m_* = 8 ± 1 mM). Part of the amino acid sequence of MsGlcNK is displayed, with the residues matching the proposed consensus sequence Q-x(2)-RE-x(2)-YA-x(3)-LP-x-W for actinobacterial glucosamine kinases highlighted in red. Download FIG S7, PDF file, 0.2 MB. Copyright © 2019 Manso et al. 2019 Manso et al. This content is distributed under the terms of the Creative Commons Attribution 4.0 International license.

## DISCUSSION

Antibiotic production in *Actinobacteria* is activated through derepression of DasR (11), a regulon of the GntR family whose activity is directly affected by GlcN-6P, the central molecule in GlcNAc metabolism (9). Two cytosolic pathways leading to GlcN-6P production have been described in *Actinobacteria*: (i) deacetylation of GlcNAc-6P, formed by phosphorylation of extracellular GlcNAc by the PTS system (22–25), and (ii) from the glycolytic intermediate fructose-6P via a GlcN-6P synthase (26, 27).

The uptake of both chitosan-derived GlcN oligosaccharides and monomeric GlcN (from chitin or from peptidoglycan turnover) by the ABC transporter CsnEFG (28) creates an alternative mechanism to obtain GlcN-6P, in which the unique family of GlcN kinases described in this work could be involved. Indeed, one proteobacterial GlcN-specific kinase has been identified in Vibrio cholerae (29) and one archaeal ADP-dependent kinase with similar efficiency for glucose and GlcN has been identified in Thermococcus kodakarensis (30) and proposed to catalyze this step in the chitinolytic pathway of these organisms. These two nonhomologous isofunctional GlcN kinases are now accompanied by the also sequence-unrelated SjGlcNK, a unique GlcN kinase from the actinomycete Streptacidiphilus jiangxiensis.

Both SjGlcNK and related orthologues were annotated as maltokinases or aminoglycoside phosphotransferases of unknown function. Despite a three-dimensional structure very similar to that of mycobacterial maltokinases, SjGlcNK was unable to phosphorylate maltose or aminoglycosides *in vitro*. The molecular determinants for substrate specificity of SjGlcNK were unveiled by the crystal structure of a productive complex with GlcN and comprise a discrete number of highly conserved residues from two short motifs, ^300^D-x-H^302^ in the catalytic loop and ^405^Q-x(2)-RE-x(2)-YA-x(3)-LP-x-W^420^ in helix α12, which we propose as consensus sequences for GlcN phosphorylation in *Actinobacteria*. In accordance, the SjGlcNK homologue from M. smegmatis, MsGlcNK, also annotated as a maltokinase and displaying the consensus sequence for GlcN binding and processing (see Table S2 in the supplemental material), was shown to phosphorylate GlcN and not maltose (Fig. S7). Interestingly, also SCO2662, a putative saccharide kinase from Streptomyces coelicolor A3(2) postulated to play a role in the phosphorylation of acquired chitosan-derived GlcN oligosaccharides (28), displays part of the GlcN-binding consensus sequence, ^255^Q-x(2)-RE-x(2)-AA^263^, although with the tyrosine residue replaced by alanine.

10.1128/mBio.00239-19.9 TABLE S2 GlcN kinases proposed as homologues of SjGlcNK in *Actinobacteria* as determined with the ConSurf server (H. Ashkenazy, S. Abadi, E. Martz, O. Chay, I. Mayrose, et al., Nucleic Acids Res, 44:W344–W350, 2016, https://doi.org/10.1093/nar/gkw408). Download Table S2, DOCX file, 0.1 MB. Copyright © 2019 Manso et al. 2019 Manso et al. This content is distributed under the terms of the Creative Commons Attribution 4.0 International license.

A search with the HMMER homologue search algorithm at the ConSurf server (48) yielded more than 80 unique sequences of uncharacterized aminoglycoside phosphotransferases or putative maltokinases from organisms of the orders *Corynebacteriales*, *Propionibacteriales*, *Frankiales*, *Streptosporangiales*, *Micromonosporales*, *Micrococcales*, and *Streptomycetales* from the phylum *Actinobacteria* (Table S2), in which the identified GlcN-contacting residues are highly conserved. It is worth noting the apparent absence of SjGlcNK homologues not just in other bacterial phyla but also in pathogenic *Actinobacteria*. Indeed, while half of the identified orthologues belong to the *Mycobacteriaceae* family, they were absent from species belonging to the recently emended genera *Mycobacterium* (including Mycobacteriumtuberculosis complex and Mycobacteriumleprae) and *Mycobacteroides* (including the rapidly growing pathogens Mycobacteroidesabscessus and Mycobacteroideschelonae), which contain the most clinically relevant mycobacterial species (49). Instead, the large majority of mycobacteria with identified orthologues were found to be *Mycolicibacterium* species or strains phylogenetically close to members of this environmental and nonpathogenic genus (49). This was further confirmed by searching the proposed consensus sequence in the nonredundant protein sequences database using BLAST, which resulted in 132 sequences exclusively from the phylum *Actinobacteria*.

While experimental evidence to elucidate the physiological role of these actinobacterial GlcN kinases is still lacking, in several cases their genomic context strongly suggests a link to secondary metabolism (Fig. 1), be it through the activation of the DasR regulon by GlcN-6P or through the putative participation in the biosynthesis of a sugar moiety that may be transferred to the aglycon of a secondary metabolite by its accompanying glycosyltransferase. These observations reinforce the hypothesis that the newly identified kinases have implications in the phosphorylation of acquired extracellular GlcN derived from the hydrolysis of chitosan, i.e., in the incorporation of exogenous GlcN into the bacterial GlcNAc metabolism.

The elucidation of the molecular determinants for GlcN phosphorylation in *Actinobacteria* presented here will be key for function assignment of a large number of uncharacterized aminoglycoside phosphotransferases and for reannotation of misannotated bacterial GlcN kinases. In addition, the structural and biochemical characterization of SjGlcNK provides new insights into the role of these unique GlcN kinases as the missing link for the incorporation of environmental GlcN to the metabolism of GlcNAc in bacteria, an important intermediate in peptidoglycan biosynthesis, glycolysis, and secondary metabolite production.

## MATERIALS AND METHODS

### Sequence analyses, genomic context, and identification of BGCs.

The SjGlcNK amino acid sequence was identified by BLAST searches using the amino acid sequences of characterized mycobacterial maltokinases (EC 2.7.1.175) and retrieved from the PATRIC (http://www.patricbrc.org/) database (genome ID 235985.3). Paralogue detection and homology between paralogues were determined based on the KEGG SSDB database (https://www.kegg.jp/kegg/ssdb). For genomic context analyses and identification of BGCs, genome assemblies of *Actinobacteria* containing SjGlcNK homologues were recovered from the NCBI database and analyzed with the *anti*biotics and *s*econdary *m*etabolite *a*nalysis *sh*ell, antiSMASH version 4.2.0 (50), using default parameters and inclusion of the ClusterFinder algorithm.

### Strains and culture conditions.

Streptacidiphilus jiangxiensis 33214 (DSM 45096), obtained from the Deutsche Sammlung von Mikroorganismen und Zellkulturen GmbH (Germany), was cultivated at 28°C for 6 days in ISP2 agar medium at pH 5.5 (4 g liter^−1^ yeast extract, 10 g liter^−1^ malt extract, 4 g liter^−1^ glucose, 20 g liter^−1^ agar). *Mycolicibacterium smegmatis* MC^2^155 (ATCC 700084), obtained from LGC Standards S.L.U. (Spain), was cultivated for 5 days at 30°C in a glycerol-based agar medium (20 g liter^−1^ glycerol, 5 g liter^−1^ Casamino Acids, 1 g liter^−1^ fumaric acid, 1 g liter^−1^ K_2_HPO_4_, 0.3 g liter^−1^ MgSO_4_, 0.02 g liter^−1^ FeSO_4_, 2 g liter^−1^ Tween 80 with pH 7.0) (51).

### Protein expression and purification.

Recombinant *S. jiangxiensis* and M. smegmatis proteins were expressed in Escherichia coli strain BL21(DE3). Cells were precultured at 37°C for 4 to 5 h in lysogeny broth (LB) supplemented with 50 mg liter^−1^ kanamycin. Six milliliters of the preculture were used to inoculate 2-liter flasks containing 750 ml of the same culture medium. When cultures reached an OD_600_ of 0.6 to 0.8 after ∼6 h of shaking at 37°C, protein production was induced by adding 500 μM isopropyl-β-d-thiogalactopyranoside, and expression proceeded for ∼16 h at 20°C. Cells were harvested by centrifugation at 4°C for 15 min at 3,500 × *g*, and the resulting pellets were resuspended in 20 mM sodium phosphate pH 7.4, 500 mM NaCl, 20 mM imidazole, and frozen at −80°C. Upon thawing, 1 mM PMSF, 1 mM DTT, and 1 mg ml^−1^ DNase were added to the cell suspension, and the cells were disrupted by sonication on ice (4 min at 25% amplitude). Cell lysates were centrifuged at 4°C for 30 min at 39,200 × *g*, and the supernatant was loaded onto a 5-ml nickel NTA agarose column (Agarose Bead Technologies) equilibrated with 20 mM sodium phosphate pH 7.4, 500 mM NaCl, 20 mM imidazole (buffer A). Bound protein was eluted with buffer A containing 500 mM imidazole. The purity of the eluted fractions was evaluated by SDS-PAGE, and those containing recombinant protein were pooled and dialyzed overnight at 4°C against 20 mM Bis-Tris propane (BTP) pH 7.4, 50 mM NaCl, 0.1 mM DTT, 0.1 mM EDTA. The protein solution was concentrated to 5 ml in a 30-kDa molecular weight cutoff centrifugal ultrafiltration device (Millipore) and loaded onto a Sephacryl S300 HR 26/60 size exclusion chromatography column (GE Healthcare), with a mobile phase adequate to the downstream use of the protein (see below). Fractions containing pure protein were combined, concentrated by ultrafiltration, flash frozen in liquid N_2_, and stored at −80°C (for crystallization experiments, the sample was used immediately). Protein concentrations were determined using the extinction coefficient at 280 nm calculated with the ProtParam tool (http://web.expasy.org/protparam/). Recombinant MvMak was produced and purified as previously described (35).

### Analysis of enzyme activity by thin-layer chromatography (TLC).

Enzyme activity was assessed in 50-μl mixtures containing 50 mM BTP pH 8.0, 10 mM MgCl_2_, 20 mM phosphate acceptor, 5 mM ATP, and 2 μg of pure recombinant enzyme at 37°C. Maltose, maltotriose, isomaltose, trehalose, turanose, leucrose, sucrose, d-mannose, 1-*O*-methylmannose, d-galactose, raffinose, d-xylose, fructose, d-tagatose, d-allose, d-glucose, l-glucose, 3-*O*-methylglucose, 2-deoxyglucose, glucuronic acid, glucosylglycerate, GlcN, GlcNAc, d-mannosamine, *N*-acetyl-d-mannosamine, d-galactosamine, *N*-acetyl-d-galactosamine, *N*-acetylmuramic acid, and the aminoglycoside antibiotics kanamycin, streptomycin, gentamicin, and hygromycin B were used as potential phosphate acceptors. Reaction mixtures were spotted on Silica 60 gel plates (Merck) and developed with one of two solvent systems: acetic acid-ethyl acetate-water-ammonia 25% (6:6:2:1, vol/vol) or ethanol-water (7:3, vol/vol). Sugars were visualized by spraying with α-naphthol–sulfuric acid solution and charring at 120°C.

### Purification of GlcN-6P and NMR analysis.

To confirm the identity of the SjGlcNK product, a 2-ml reaction mixture (50 mM BTP pH 8.0, 20 mM MgCl_2_, 20 mM GlcN, 10 mM ATP, and 120 μg SjGlcNK) was incubated overnight at 37°C and separated by TLC. The mixture was spotted on Silica 60 gel plates (Merck) and developed with a methanol-chloroform-acetic acid-water (4:1:1:1, vol/vol) solvent system. The marginal lanes of the TLC plates were stained to identify the spot corresponding to the SjGlcNK product, and the remaining unstained lanes were scrapped in the same region. The product was extracted from the silica gel with ultrapure water, lyophilized, and further purified on a Sephadex G10 column (GE Healthcare) with a water flow of 1 ml min^−1^, followed by a second lyophilization step before NMR analysis. All NMR spectra were acquired on an Avance III 400 spectrometer from Bruker operating at a central proton frequency of 400.13 MHz equipped with a BBI(F)-z H-X-D (5-mm) probe at 25°C with presaturation of the water signal.

### Data availability.

The X-ray diffraction images (https://doi.org/10.15785/SBGRID/614, https://doi.org/10.15785/SBGRID/615, and https://doi.org/10.15785/SBGRID/616) were deposited with the Structural Biology Data Grid (52). Coordinates and structure factors were deposited at the Protein Data Bank (PDB) under accession numbers 6HWJ (SjGlcNK, crystal form A), 6HWK (SjGlcNK, crystal form B), and 6HWL (SjGlcNK-GlcN-ADP-P_i_ complex). SAXS data were deposited at the Small Angle Scattering Biological Data Bank (SASBDB) (53) under codes SASDEL6, SASDEM6, SASDEN6, SASDEP6, SASDEQ6, and SASDER6. Other data are available from the corresponding authors upon reasonable request.

Additional methods are described in Text S1 in the supplemental material.

10.1128/mBio.00239-19.9 TEXT S1 Supplemental methods. Download Text S1, DOCX file, 0.1 MB. Copyright © 2019 Manso et al. 2019 Manso et al. This content is distributed under the terms of the Creative Commons Attribution 4.0 International license.
