# Supplementary material for: Molecular Fingerprints for a Novel Enzyme Family in Actinobacteria with Glucosamine Kinase Activity
Source: mBio. 2019 May 14;10(3):e00239-19. doi: 10.1128/mBio.00239-19 (PMC6520443; doi:10.1128/mBio.00239-19)
Supplement: FIG S1 [file mBio.00239-19-sf001.pdf]

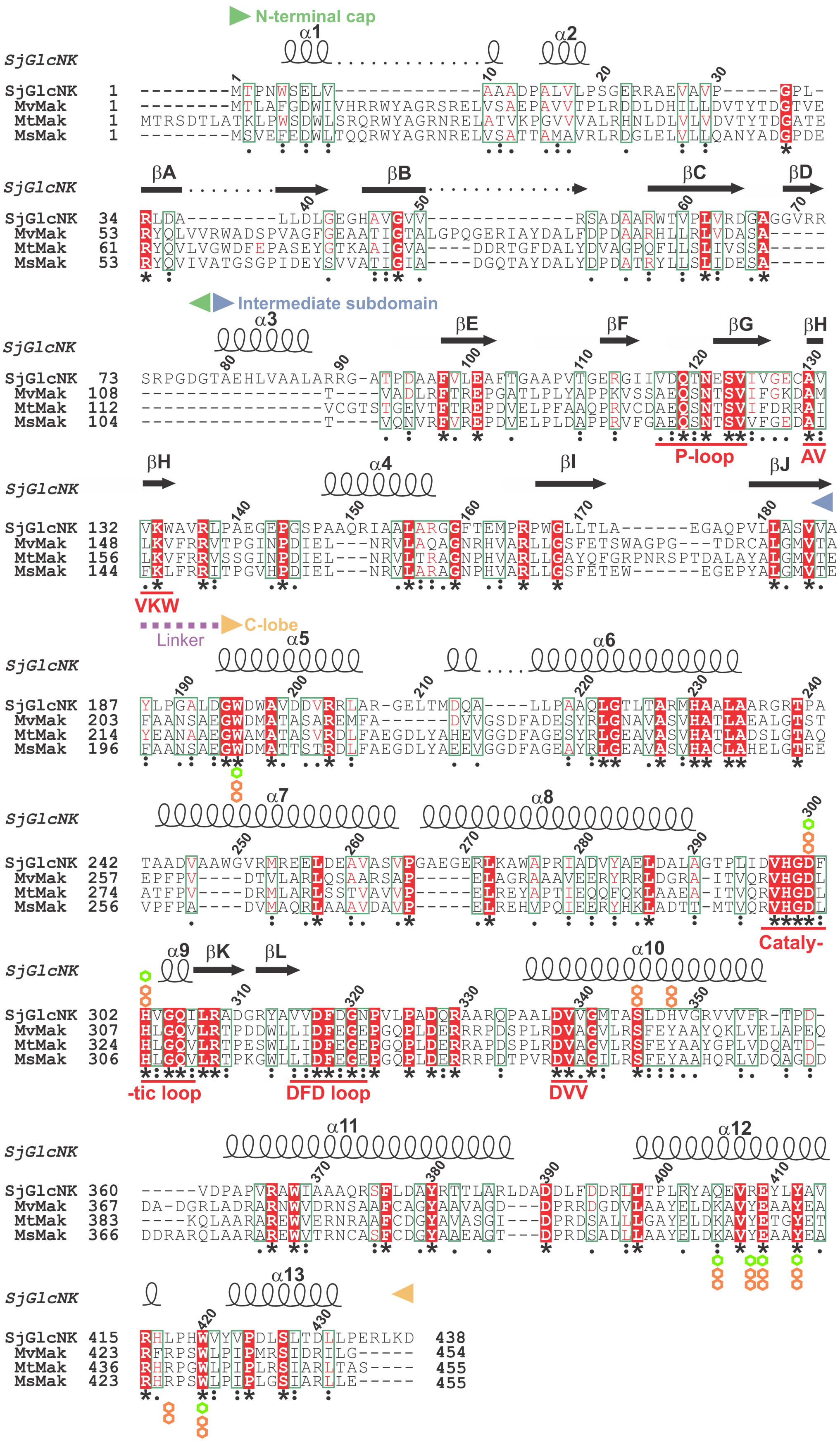

N-terminal cap
Intermediate subdomain
C-lobe
Linker
P-loop
AV
VKW
Catalytic loop
DFD loop
DVV
SjGlcNK
MvMak
MtMak
MsMak

Fig. S1. Multiple amino acid sequence alignment of SjGlcNK with homologous mycobacterial maltokinases. The amino acid sequence of SjGlcNK from *Streptacidiphilus jiangxiensis* (UniProtKB entry A0A1H7TQR5) was aligned with those of MvMak from *Mycolicibacterium vanbaalenii* (UniProtKB entry A1TH50), MtMak from *Mycobacterium tuberculosis* (UniProtKB entry O07177), and MsMak from *Mycolicibacterium smegmatis* (UniProtKB entry A0R6D9). Strictly conserved alignment positions are shown in inverted type on a red background. Secondary structure elements for SjGlcNK are represented above the alignment. The catalytic and DFD loops, the P-loop, and the DVV and AVVKW motifs are labeled in red. The residues that participate in GlcN and maltose binding are indicated by green and orange hexagons, respectively.
