# Supplementary material for: Molecular Fingerprints for a Novel Enzyme Family in Actinobacteria with Glucosamine Kinase Activity
Source: mBio. 2019 May 14;10(3):e00239-19. doi: 10.1128/mBio.00239-19 (PMC6520443; doi:10.1128/mBio.00239-19)
Supplement: FIG S2 [file mBio.00239-19-sf002.pdf]

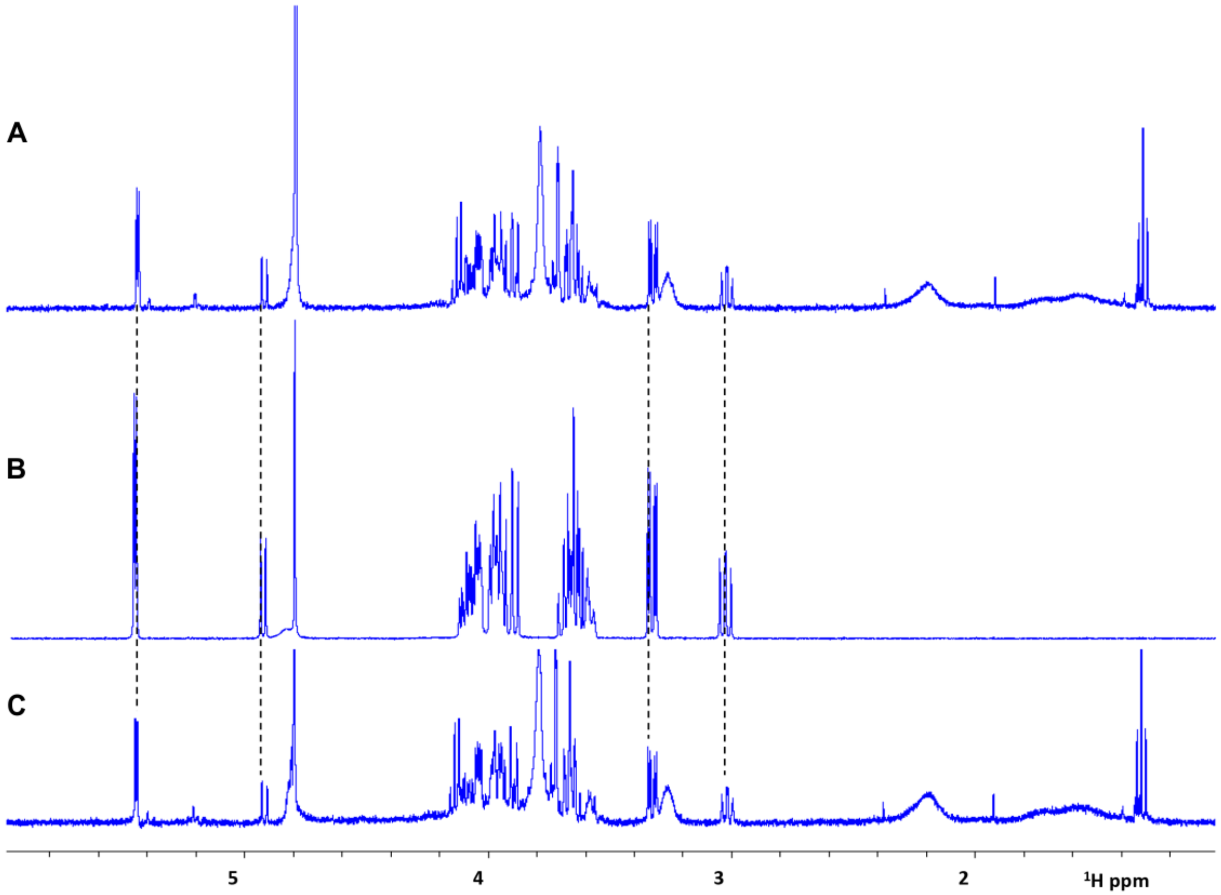

Fig. S2. 1H-NMR spectra of the enzymatic product of SjGlcNK. (**A**) Sample from enzymatic reaction mixture purified by thin-layer chromatography; (**B**) GlcN-6P standard (Sigma-Aldrich) in D_2O; (**C**) Sample from **A** spiked with an aliquot of standard **B**. The alignment between the signals in samples **A** and **B** is perfect. The spiking of sample **A** with the standard revealed no new signals and a slight increase of those already present in the sample, thus confirming the identity of the sample compound as GlcN-6P.
