# Supplementary material for: Molecular Fingerprints for a Novel Enzyme Family in Actinobacteria with Glucosamine Kinase Activity
Source: mBio. 2019 May 14;10(3):e00239-19. doi: 10.1128/mBio.00239-19 (PMC6520443; doi:10.1128/mBio.00239-19)
Supplement: FIG S3 [file mBio.00239-19-sf003.pdf]

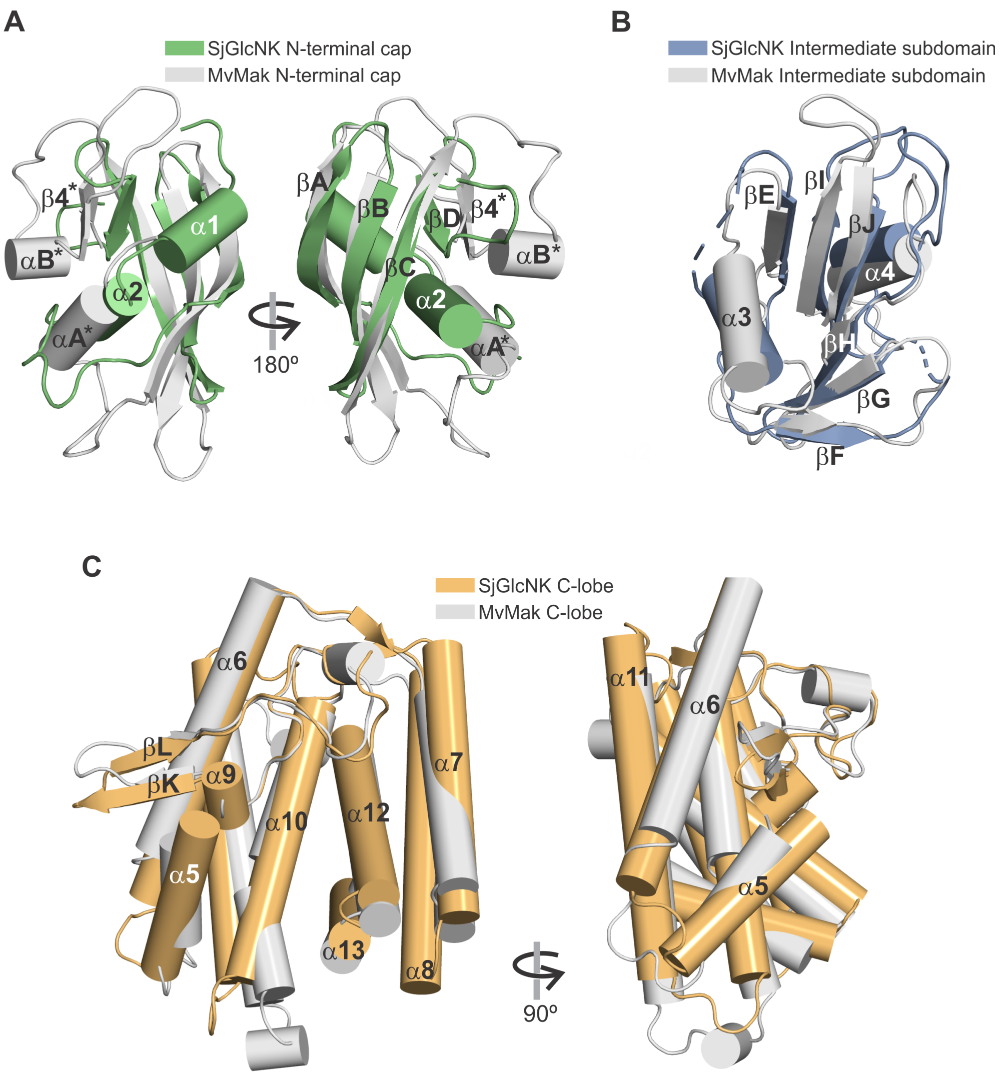

Fig. S3. Overall structure of SjGlcNK and structural comparison with MvMak. (**A**) Structural superposition of SjGlcNK (colored as in Fig. 2F) and MvMak (gray; PDB entry 4U94 (J. Fraga, A. Maranha, V. Mendes, P. J. B. Pereira, N. Empadinhas, and S. Macedo-Ribeiro, Sci Rep 5:8026, 2015, doi:10.1038/srep08026)) N-terminal caps (two views rotated by 180° around *y*), (**B**) intermediate subdomains, and (**C**) C-lobes (two views rotated by 90° around *y*). Secondary structure elements are labeled.
