# Supplementary material for: Molecular Fingerprints for a Novel Enzyme Family in Actinobacteria with Glucosamine Kinase Activity
Source: mBio. 2019 May 14;10(3):e00239-19. doi: 10.1128/mBio.00239-19 (PMC6520443; doi:10.1128/mBio.00239-19)
Supplement: FIG S4 [file mBio.00239-19-sf004.pdf]

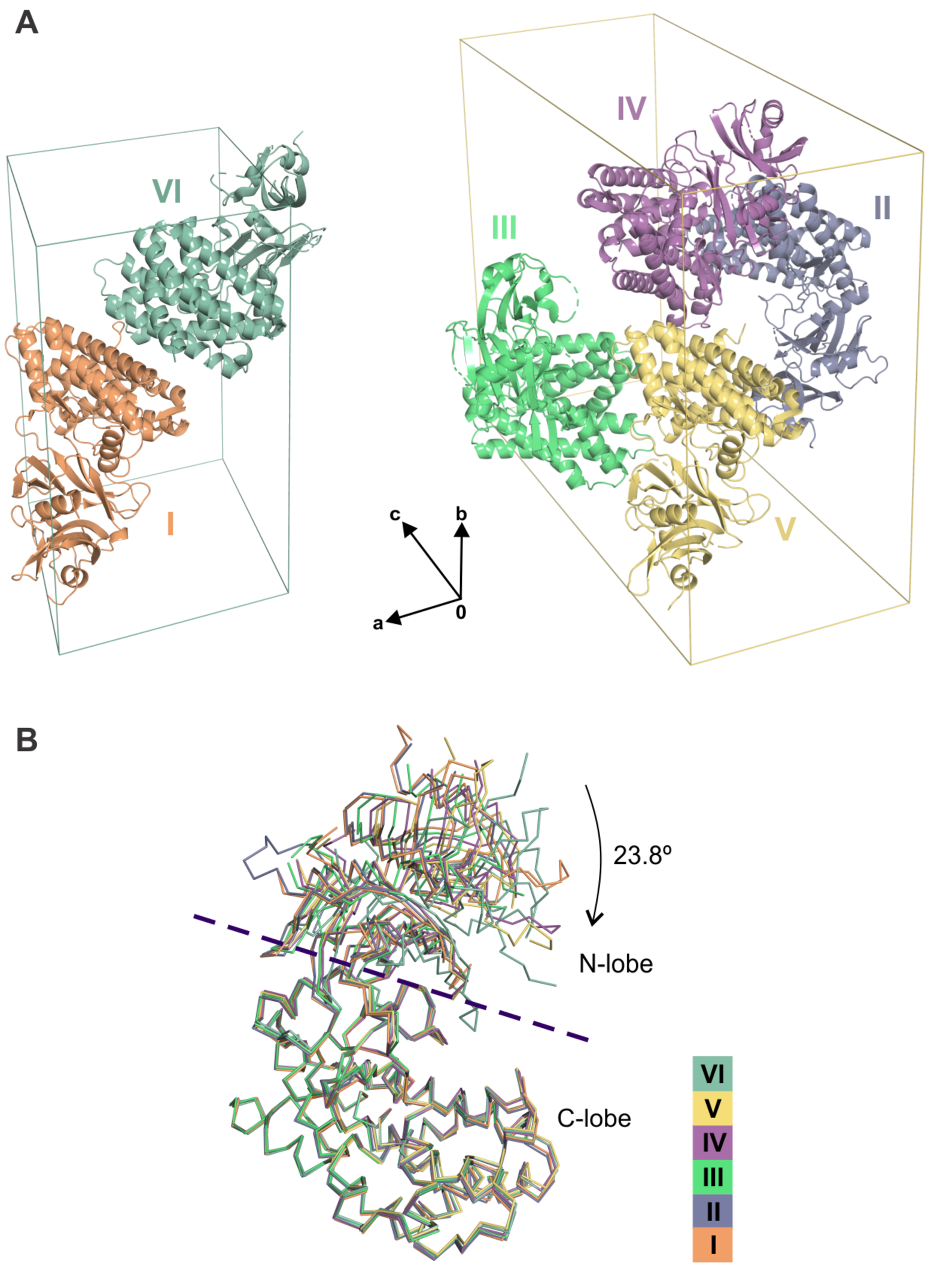

Fig. S4. The two crystallographic forms, A and B, of SjGlcNK comprise six conformational states of the enzyme. (**A**) Cartoon representation of two (left, crystal form A) and four molecules (right, crystal form B) found in the asymmetric units of the two SjGlcNK crystal forms. The individual structures are labeled with Roman numerals. (**B**) Superposition of the Cα traces of the six SjGlcNK monomers (colored as in **A**), by alignment of the C-lobe subdomains. The

orientations of the N-lobe in the open and closed conformations are related by a rotation of 23.8° around a hinge axis (dashed line) as determined by DynDom (S. Hayward and H. J. Berendsen, Proteins, 30:144-154, 1998, doi:10.1002/(SICI)1097-0134(19980201)30:2<144::AID-PROT4>3.0.CO;2-N).
