# Supplementary material for: Molecular Fingerprints for a Novel Enzyme Family in Actinobacteria with Glucosamine Kinase Activity
Source: mBio. 2019 May 14;10(3):e00239-19. doi: 10.1128/mBio.00239-19 (PMC6520443; doi:10.1128/mBio.00239-19)
Supplement: FIG S5 [file mBio.00239-19-sf005.pdf]

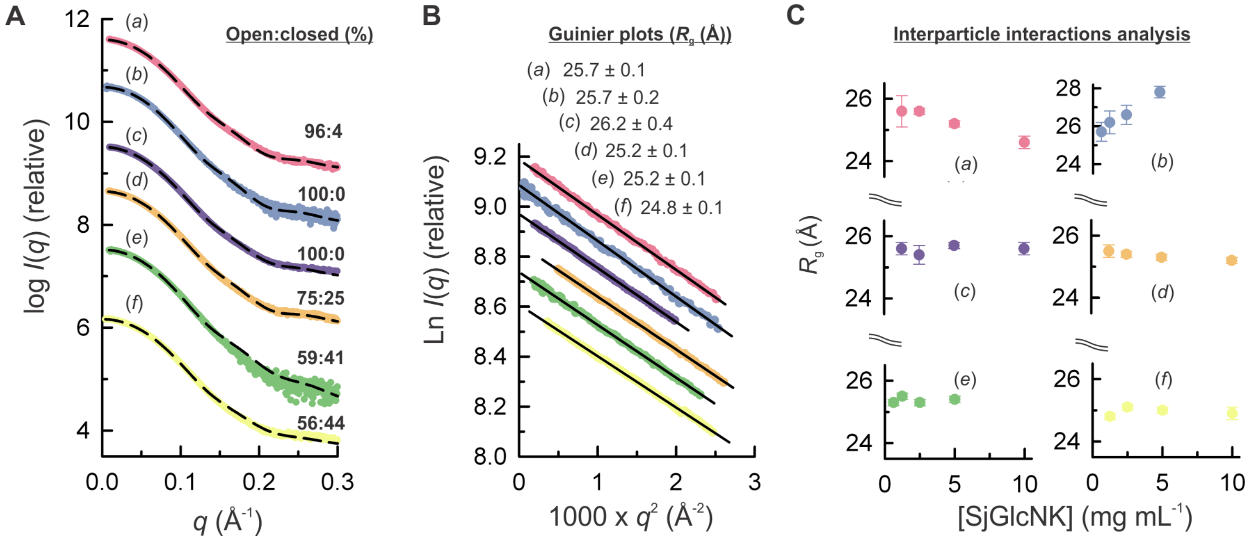

Fig. S5. Ligand-induced conformational transformation of SjGlcNK in solution probed by SAXS. (**A**) SAXS profiles extrapolated to infinite dilution of SjGlcNK (a) in absence of substrates (red) and in presence of (b) 200 m*M* GlcN (blue), (c) 50 m*M* glucose (violet), (d) 1 m*M* ATP (orange), (e) 200 m*M* GlcN and 1m*M* ATP (green), and (f) 50 m*M* glucose and 1 m*M* ATP (yellow). The curves are offset on the log scale. The scattering calculated for a combination of open and closed conformations (ratio given above the curves), is shown as dashed lines. (**B**) Guinier plots of the scattering data shown in A. R_g values (± SD) are indicated for each experiment. (**C**) Plot of Guinier R_g at several concentrations, for the same combinations of protein and ligand(s) as in **A**.
