# Supplementary material for: Molecular Fingerprints for a Novel Enzyme Family in Actinobacteria with Glucosamine Kinase Activity
Source: mBio. 2019 May 14;10(3):e00239-19. doi: 10.1128/mBio.00239-19 (PMC6520443; doi:10.1128/mBio.00239-19)
Supplement: TABLE S1 [file mBio.00239-19-st001.docx]

**Table S1. Small angle X-ray scattering results for SjGlcNK with and without ligands*^a^***

(***a*) Sample details.**

| Organism | *Streptacidiphilus jiangxiensis* |
| --- | --- |
| Source | Expressed in *E. coli* BL21 (DE3) |
| UniProtKB entry (residues in construct; C-terminal 6×His-tag residues) | A0A1H7TQR5 (1-451; 439-451) |
| Extinction coefficient [*A*_280_, 0.1% (*w*/*v*)] | 1.387 |
|  from chemical composition (cm^3^ g^-1^) | 0.742 |
| Particle contrast from sequence and solvent constituents, Δ** (*ρ*_protein_ - *ρ*_solvent_; 10^10^ cm^-2^) | 2.75 (12.22 - 9.46) |
| *M* from chemical composition (Da) | 48,249.8 |
| Solvent (solvent blanks taken from SEC flow-through prior to elution of protein) | 20 m*M* Tris-HCl pH 8.0, 150 m*M* NaCl, 10 m*M* MgCl_2_, 5 m*M* DTT |

**(*b*) SAXS data collection.**

| Instrument | ESRF BM29 [7] |
| --- | --- |
| Detector | Dectris PILATUS 1M |
| Wavelength (Å) | 0.9919 |
| Beam size (μm) | 700 × 700 |
| Camera length (m) | 2.867 |
| *q* measurement range (Å^-1^) | 0.0035-0.5 |
| Absolute scaling method | Comparison with scattering from pure H_2_O |
| Normalization | To transmitted intensity by beam-stop counter |
| Monitoring for radiation damage | Data frame-by-frame comparison |
| Exposure time (s) | 10 × 1 |
| Sample configuration | Standard 'batch' mode |
| Sample temperature (ºC) | 10 |

**(*c*) SAXS structural parameters and atomistic modelling.**

|  | SjGlcNK | SjGlcNK  + 200 m*M* GlcN | SjGlcNK  + 1 m*M* ATP | SjGlcNK  + 200 m*M* GlcN  + 1 m*M* ATP | SjGlcNK  + 50 m*M* Glucose | SjGlcNK  + 50 m*M* Glucose  + 1 m*M* ATP |
| --- | --- | --- | --- | --- | --- | --- |
| **Structural parameters** |  |  |  |  |  |  |
| Guinier analysis |  |  |  |  |  |  |
| *I*(0)/c (10^-2^ cm^2^ mg^-1^)*^b^* | 3.280 ± 0.004 | 2.671 ± 0.004 | 3.672 ± 0.002 | 2.637 ± 0.004 | 2.977 ± 0.004 | 3.026 ± 0.002 |
| *R*_g_ (Å) | 25.7 ± 0.1 | 25.7 ± 0.1 | 25.2 ± 0.1 | 25.2 ± 0.1 | 26.2 ± 0.4 | 24.8 ± 0.1 |
| *q*_min_ (Å^-1^) | 0.014 | 0.007 | 0.022 | 0.014 | 0.028 | 0.019 |
| *qR*_g_ max | 1.29 | 1.29 | 1.29 | 1.21 | 1.30 | 1.23 |
| Correlation coefficient, *R*^2^ | 0.999 | 0.997 | 0.999 | 0.998 | 0.999 | 0.999 |
| *M* from *I*(0)/c  (ratio to predicted)*^c^* | 45,226 (0.94) | 36,829 (0.76) | 50,659 (1.05) | 36,360 (0.75) | 41,047 (0.85) | 41,724 (0.86) |
| *P*(*r*) analysis |  |  |  |  |  |  |
| *I*(0)/c (10^-2^ cm^2^ mg^-1^) | 3.283 ± 0.001 | 2.684 ± 0.001 | 3.667 ± 0.002 | 2.640 ± 0.001 | 2.962 ± 0.001 | 3.031 ± 0.001 |
| *R*_g_ (Å) | 25.9 ± 0.1 | 26.0 ± 0.2 | 25.2 ± 0.2 | 25.4 ± 0.2 | 26.1 ± 0.2 | 24.9 ± 0.2 |
| *d*_max_ (Å) | 80 | 80 | 78 | 77 | 80 | 77 |
| *q* range (Å^-1^) | 0.00913-0.30123 | 0.0090-0.3012 | 0.0223-0.3092 | 0.0137-0.2951 | 0.0279-0.3054 | 0.0190-0.3224 |
| Total estimate from *GNOM* | 0.60 | 0.67 | 0.60 | 0.70 | 0.60 | 0.61 |
| *M* from *I*(0)  (ratio to predicted value) | 45,267 (0.94) | 37,098 (0.77) | 50,562 (1.05) | 36,400 (0.75) | 40,840 (0.85) | 41,791 (0.87) |
| Porod volume (Å^-3^)  (ratio *V*_P_ / calculated *M*) | 72,000 (1.59) | 78,290 (2.10) | 72,950(1.44) | 75,800 (2.08) | 73,250 (1.79) | 73,100 (1.75) |
| **Atomistic modeling** |  |  |  |  |  |  |
| Contribution (%) of the closed conformation (VI) to the overall scattering as determined with *OLIGOMER*^d^ | 4 | 0 | 25 | 41 | 0 | 44 |
| SASBDB code^e^ | SASDEL6 | SASDEM6 | SASDEN6 | SASDEP6 | SASDEQ6 | SASDER6 |

*^a^*Description of the accuracy and confidence in the SAXS data and modelling outputs are reported following the *2017 publication guidelines and recommendations for solution small-angle scattering data* (J. Trewhella, A. P. Duff, D. Durand, F. Gabel, J. M. Guss, W. A. Hendrickson, G. L. Hura, D. A. Jacques, N. M. Kirby, A. H. Kwan, J. Pérez, L. Pollack, T. M. Ryan, A. Sali, D. Schneidman-Duhovny, T. Schwede, D. I. Svergun, M. Sugiyama, J. A. Tainer, P. Vachette, J. Westbrook, and A. E. Whitten, Acta Crystallogr D Biol Crystallogr, 73:710-728, 2017, doi:10.1107/S2059798317011597). *^b^*Absolute intensities were determined using water as secondary standard. *^c^M* was calculated as [*N*_A_ *I*(0)/*c*]/Δ*ρ*_M_^2^, where *I*(0)/*c* is the forward scattering normalized against concentration, Δ*ρ*_M_ = [*ρ*_M,prot_ - (*ρ*_solv_)]*r*_o_ is the scattering contrast per mass, *N*_A_ = 6.023 × 10^23^ mol^-1^ is the Avogadro number, *ρ*_M,prot_ = 3.22 × 10^23^ e g^-1^ is the number of electrons per mass of dry protein, *ρ*_solv_ = 3.34 × 10^23^ e cm^-3^ is the number of electrons per volume of the aqueous solvent, is the partial specific volume of the protein and *r*_o_ = 2.8179 × 10^-13^ cm is the scattering length of an electron (L. A. Feigin and D. I. Svergun, Structure Analysis by Small-Angle X-Ray and Neutron Scattering, Springer US, 1987, [www.springer.com/la/book/9781475766264](http://www.springer.com/la/book/9781475766264), E. Mylonas and D. I. Svergun, J Appl Crystallogr, 40:s245–s249, 2007, doi:10.1107/S002188980700252X, D. Orthaber, A. Bergmann, and O. Glatter, J Appl Crystallogr, 33:218–225, 2000, doi:10.1107/S0021889899015216). *^d^*Form factors for *OLIGOMER* included the calculated intensities for the open (I) and closed (VI) conformations with *CRYSOL*. *^e^*SASBDB, Small Angle Scattering Biological Data Bank (E. Valentini, A. G. Kikhney, G. Previtali, C. M. Jeffries, and D. I. Svergun, Nucleic Acids Res, 43:D357-D363, 2015, doi:10.1093/nar/gku1047).
