# Supplementary material for: Molecular Fingerprints for a Novel Enzyme Family in Actinobacteria with Glucosamine Kinase Activity
Source: mBio. 2019 May 14;10(3):e00239-19. doi: 10.1128/mBio.00239-19 (PMC6520443; doi:10.1128/mBio.00239-19)
Supplement: FIG S6 [file mBio.00239-19-sf006.pdf]

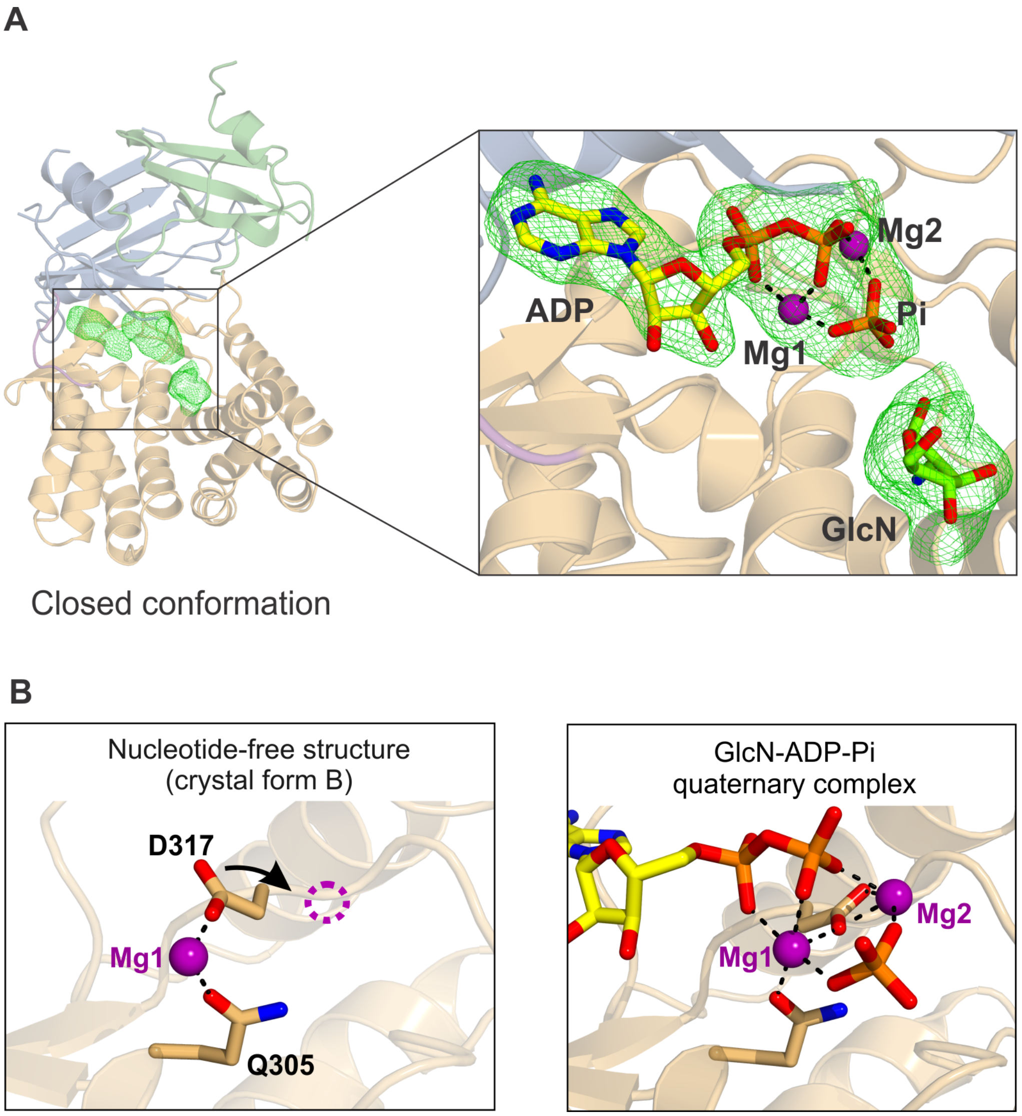

Fig. S6. Crystal structure of the complex between SjGlcNK, GlcN and ATP. (**A**) The structure of the complex reveals the transition state of the phosphoryl transfer reaction of ATP to GlcN. Although a residual, non-interpretable positive electron density was observed near the active site of the closed state (VI in Fig. 3A) of apo-SjGlcNK (crystal form A), in crystals obtained by co-crystallization with a molar excess of ATP and GlcN (Table 1) the substrates could be easily located in the electron density map. The structure of SjGlcNK (closed conformation) is shown in ribbon representation colored as in Fig. 2F. The Polder mF_o-DF_c map (D. Liebschner, P. V. Afonine, N. W. Moriarty, B. K. Poon, O. V. Sobolev, T. C. Terwilliger, and

P. D. Adams, Acta Crystallogr D Biol Crystallogr, 73:148-157, 2017, doi:10.1107/S2059798316018210) (contoured at 4σ) around the ADP, inorganic phosphate (Pi), magnesium ions (Mg1 and Mg2) and GlcN is shown as a green mesh. (**B**) Ligand binding induces the reorientation of the side chain of D317. Magnesium binding sites are shown for the nucleotide-free SjGlcNK structure (left) and for the phosphorylation transition state (right) found in the GlcN-ADP-Pi quaternary complex.
