# Supplementary material for: Molecular Fingerprints for a Novel Enzyme Family in Actinobacteria with Glucosamine Kinase Activity
Source: mBio. 2019 May 14;10(3):e00239-19. doi: 10.1128/mBio.00239-19 (PMC6520443; doi:10.1128/mBio.00239-19)
Supplement: FIG S7 [file mBio.00239-19-sf007.pdf]

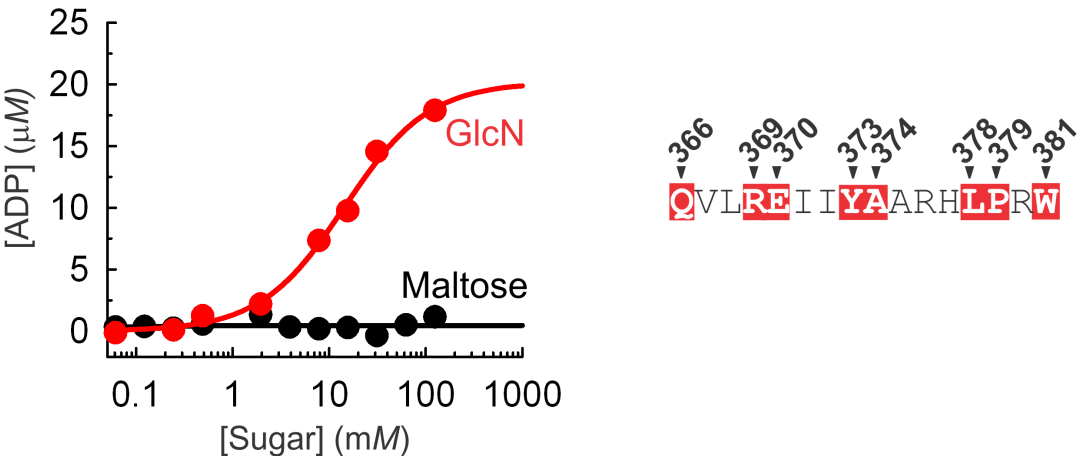

Fig. S7. The putative maltokinase from *M. smegmatis*, MsGlcNK, phosphorylates GlcN. Similar to SjGlcNK, MsGlcNK displays preference for GlcN over maltose as substrate. The enzymatic activity was measured as ADP release from ATP using the ADP-Glo™ kinase Assay Kit (Promega) (H. Zegzouti, M. Zdanovskaia, K. Hsiao, and S. A. Goueli, Assay Drug Dev Technol, 7:560–572, 2009, doi:10.1089/adt.2009.0222). Kinase reactions were performed in 100 m*M* Tris-HCl pH 7.5, 20 m*M* $MgCl_2$, 0.1 mg mL^{-1} BSA with [MsGlcNK] = 1 μ*M*, [ATP] = 2.5 m*M* and varying concentrations of GlcN (red dots) and maltose (black dots), upon incubation at RT for 5 min. Fitting the data to the Michaelis-Menten equation (red line) resulted in a K_m of 14 ± 2 m*M*, very similar to that of SjGlcNK (K_m = 8 ± 1 m*M*). Part of the amino acid sequence of MsGlcNK is displayed, with the residues matching the proposed consensus sequence Q-x(2)-RE-x(2)-YA-x(3)-LP-x-W for actinobacterial glucosamine kinases highlighted in red.
