# Supplementary material for: Molecular Fingerprints for a Novel Enzyme Family in Actinobacteria with Glucosamine Kinase Activity
Source: mBio. 2019 May 14;10(3):e00239-19. doi: 10.1128/mBio.00239-19 (PMC6520443; doi:10.1128/mBio.00239-19)
Supplement: TABLE S2 [file mBio.00239-19-st002.docx]

**Table S2. GlcN kinases proposed as homologues of SjGlcNK in *Actinobacteria* as determined with the ConSurf server (H. Ashkenazy, S. Abadi, E. Martz, O. Chay, I. Mayrose, T. Pupko, and N. Ben-Tal, Nucleic Acids Res, 44:W344-W350, 2016, doi:10.1093/nar/gkw408).**

| Organism | Lineage | Sequence | Protein name | AntiSMASH |
| --- | --- | --- | --- | --- |
| *Mycobacterium grossiae* | *Actinobacteria*  *Actinobacteria*  *Corynebacteriales*  *Mycobacteriaceae*  *Mycobacterium* | UniRef90_A0A1E8Q961 | Aminoglycoside phosphotransferase | No cluster detected |
| *Mycolicibacterium litorale* | *Actinobacteria*  *Actinobacteria*  *Corynebacteriales*  *Mycobacteriaceae*  *Mycolicibacterium* | UniRef90_A0A1U9PBG4 | Aminoglycoside phosphotransferase | Type I PKS / NRPS cluster |
| *Mycolicibacterium chlorophenolicum* | *Actinobacteria*  *Actinobacteria*  *Corynebacteriales*  *Mycobacteriaceae*  *Mycolicibacterium* | UniRef90_A0A0J6VYP0 | Maltokinase | NRPS cluster |
| *Mycolicibacterium bacteremicum* | *Actinobacteria*  *Actinobacteria*  *Corynebacteriales*  *Mycobacteriaceae*  *Mycolicibacterium* | UniRef90_A0A1W9YXQ7 | Aminoglycoside phosphotransferase | Putative cluster |
| *Mycobacterium* sp*.* (strain CECT 8779) | *Actinobacteria*  *Actinobacteria*  *Corynebacteriales*  *Mycobacteriaceae*  *Mycobacterium* | UniRef90_UPI000BFECE6E | Aminoglycoside phosphotransferase | Putative cluster |
| *Mycolicibacterium iranicum* | *Actinobacteria*  *Actinobacteria*  *Corynebacteriales*  *Mycobacteriaceae*  *Mycolicibacterium* | UniRef90_A0A1X1WWQ4 | Aminoglycoside phosphotransferase | No cluster detected |
| Uncultured *Mycobacterium* sp*.* | *Actinobacteria*  *Actinobacteria*  *Corynebacteriales*  *Mycobacteriaceae*  *Mycobacterium* | UniRef90_A0A1Y5PAA8 | Putative 1,4-alpha-glucan branching enzyme | Type I PKS / NRPS cluster |
| *Mycolicibacterium wolinskyi* | *Actinobacteria*  *Actinobacteria*  *Corynebacteriales*  *Mycobacteriaceae*  *Mycolicibacterium* | UniRef90_A0A132PT41 | Aminoglycoside phosphotransferase | Putative cluster |
| *Mycobacterium* sp. (strain 3519A) | *Actinobacteria*  *Actinobacteria*  *Corynebacteriales*  *Mycobacteriaceae*  *Mycobacterium* | UniRef90_UPI000C7E100F | Aminoglycoside phosphotransferase | Putative cluster |
| *Mycobacterium* sp*.* (strain ACS1612) | *Actinobacteria*  *Actinobacteria*  *Corynebacteriales*  *Mycobacteriaceae*  *Mycobacterium* | UniRef90_A0A1A1YYA9 | Aminoglycoside phosphotransferase | No cluster detected |
| *Mycobacterium* sp*.* (strain WY10) | *Actinobacteria*  *Actinobacteria*  *Corynebacteriales*  *Mycobacteriaceae*  *Mycobacterium* | UniRef90_A0A1J0UBQ9 | Aminoglycoside phosphotransferase | Type I PKS / NRPS cluster |
| *Mycolicibacterium rufum* | *Actinobacteria*  *Actinobacteria*  *Corynebacteriales*  *Mycobacteriaceae*  *Mycolicibacterium* | UniRef90_A0A099CJH1 | Aminoglycoside phosphotransferase | NRPS cluster |
| *Mycolicibacterium rhodesiae* | *Actinobacteria*  *Actinobacteria*  *Corynebacteriales*  *Mycobacteriaceae*  *Mycolicibacterium* | UniRef90_A0A1X0J1N8 | Aminoglycoside phosphotransferase | Type I PKS / NRPS cluster |
| *Mycolicibacterium obuense* | *Actinobacteria*  *Actinobacteria*  *Corynebacteriales*  *Mycobacteriaceae*  *Mycolicibacterium* | UniRef90_A0A0J6VTN0 | Maltokinase | NRPS cluster |
| *Mycolicibacterium smegmatis* | *Actinobacteria*  *Actinobacteria*  *Corynebacteriales*  *Mycobacteriaceae*  *Mycolicibacterium* | UniRef90_A0A0D6IZ29 | Trehalose synthase-fused maltokinase | No cluster detected |
| *Mycolicibacterium aromaticivorans* (strain JS19b1) | *Actinobacteria*  *Actinobacteria*  *Corynebacteriales*  *Mycobacteriaceae*  *Mycolicibacterium* | UniRef90_A0A064CHT8 | Aminoglycoside phosphotransferase | Type I PKS / NRPS cluster |
| *Mycobacterium* sp*.* (strain shizuoka-1) | *Actinobacteria*  *Actinobacteria*  *Corynebacteriales*  *Mycobacteriaceae*  *Mycobacterium* | UniRef90_A0A2C9T723 | Uncharacterized protein | Putative cluster |
| *Mycobacterium* sp*.* (strain UNC267MFSha1.1M11) | *Actinobacteria*  *Actinobacteria*  *Corynebacteriales*  *Mycobacteriaceae*  *Mycobacterium* | UniRef90_A0A1G4V8L9 | Maltokinase | NRPS cluster |
| *Mycobacterium goodii* | *Actinobacteria*  *Actinobacteria*  *Corynebacteriales*  *Mycobacteriaceae*  *Mycolicibacterium* | UniRef90_A0A0K0X5X8 | Aminoglycoside phosphotransferase | No cluster detected |
| *Mycolicibacterium neoaurum* | *Actinobacteria*  *Actinobacteria*  *Corynebacteriales*  *Mycobacteriaceae*  *Mycolicibacterium* | UniRef90_A0A024QK26 | Trehalose synthase-fused maltokinase | Putative cluster |
| *Mycobacterium* sp*.* (strain Soil538) | *Actinobacteria*  *Actinobacteria*  *Corynebacteriales*  *Mycobacteriaceae*  *Mycobacterium* | UniRef90_A0A0Q9JII5 | Aminoglycoside phosphotransferase | Putative cluster |
| *Mycobacterium* sp. (strain M26) | *Actinobacteria*  *Actinobacteria*  *Corynebacteriales*  *Mycobacteriaceae*  *Mycobacterium* | UniRef90_UPI00073E1503 | Hypothetical protein | No cluster detected |
| *Mycolicibacterium agri* | *Actinobacteria*  *Actinobacteria*  *Corynebacteriales*  *Mycobacteriaceae*  *Mycolicibacterium* | UniRef90_A0A2A7NDE4 | Aminoglycoside phosphotransferase | Putative cluster |
| *Mycolicibacterium neoaurum* (strain VKM Ac-1815D) | *Actinobacteria*  *Actinobacteria*  *Corynebacteriales*  *Mycobacteriaceae*  *Mycolicibacterium* | UniRef90_V5X7B3 | Aminoglycoside phosphotransferase | No cluster detected |
| *Mycobacterium* sp*.* (strain MS1601) | *Actinobacteria*  *Actinobacteria*  *Corynebacteriales*  *Mycobacteriaceae*  *Mycobacterium* | UniRef90_A0A1P8XBH9 | Aminoglycoside phosphotransferase | No cluster detected |
| *Mycolicibacterium vaccae* (strain ATCC 25954) | *Actinobacteria*  *Actinobacteria*  *Corynebacteriales*  *Mycobacteriaceae*  *Mycolicibacterium* | UniRef90_K0UL61 | Aminoglycoside phosphotransferase | No cluster detected |
| *Mycobacterium* sp*.* (strain Root135) | *Actinobacteria*  *Actinobacteria*  *Corynebacteriales*  *Mycobacteriaceae*  *Mycobacterium* | UniRef90_A0A0T1W453 | Aminoglycoside phosphotransferase | No cluster detected |
| *Mycolicibacterium brisbanense* | *Actinobacteria*  *Actinobacteria*  *Corynebacteriales*  *Mycobacteriaceae*  *Mycolicibacterium* | UniRef90_A0A117I5S0 | Aminoglycoside phosphotransferase | Putative cluster |
| *Mycobacterium* sp*.* (strain 852013-50091_SCH5140682) | *Actinobacteria*  *Actinobacteria*  *Corynebacteriales*  *Mycobacteriaceae*  *Mycobacterium* | UniRef90_A0A1A0XUC1 | Aminoglycoside phosphotransferase | No cluster detected |
| *Mycobacterium* sp*.* (strain djl-10) | *Actinobacteria*  *Actinobacteria*  *Corynebacteriales*  *Mycobacteriaceae*  *Mycobacterium* | UniRef90_A0A1B1WSU8 | Aminoglycoside phosphotransferase | No cluster detected |
| *Mycobacterium* sp*.*(strain ITM-2016-00317) | *Actinobacteria*  *Actinobacteria*  *Corynebacteriales*  *Mycobacteriaceae*  *Mycolicibacterium* | UniRef90_A0A2S8M089 | Aminoglycoside phosphotransferase | No cluster detected |
| *Mycobacterium* sp*.* (strain GA-2829) | *Actinobacteria*  *Actinobacteria*  *Corynebacteriales*  *Mycobacteriaceae*  *Mycobacterium* | UniRef90_A0A101B6W8 | Aminoglycoside phosphotransferase | No cluster detected |
| *Mycolicibacterium aurum* | *Actinobacteria*  *Actinobacteria*  *Corynebacteriales*  *Mycobacteriaceae*  *Mycolicibacterium* | UniRef90_UPI00065E7868 | Aminoglycoside phosphotransferase | NRPS cluster |
| *Mycobacterium dioxanotrophicus* | *Actinobacteria*  *Actinobacteria*  *Corynebacteriales*  *Mycobacteriaceae*  *Mycobacterium* | UniRef90_A0A1Y0C060 | Aminoglycoside phosphotransferase | Putative cluster |
| *Mycobacterium* sp*.* (strain Root265) | *Actinobacteria*  *Actinobacteria*  *Corynebacteriales*  *Mycobacteriaceae*  *Mycobacterium* | UniRef90_A0A0Q9B0V8 | Aminoglycoside phosphotransferase | Putative cluster |
| *Mycolicibacterium diernhoferi* | *Actinobacteria*  *Actinobacteria*  *Corynebacteriales*  *Mycobacteriaceae*  *Mycolicibacterium* | UniRef90_A0A1Q4H776 | Aminoglycoside phosphotransferase | Putative cluster |
| *Nocardioides lianchengensis* | *Actinobacteria*  *Actinobacteria*  *Propionibacteriales*  *Nocardioidaceae*  *Nocardiodes* | UniRef90_A0A1G6VCL7 | Maltokinase | Putative cluster |
| *Mycobacterium* sp*.* (strain NAZ190054) | *Actinobacteria*  *Actinobacteria*  *Corynebacteriales*  *Mycobacteriaceae*  *Mycobacterium* | UniRef90_A0A132T811 | Aminoglycoside phosphotransferase | Putative cluster |
| *Frankia* sp*.* (strain EUN1h) | *Actinobacteria*  *Actinobacteria*  *Frankiales*  *Frankiaceae*  *Frankia* | UniRef90_A0A1S1QHA0 | Aminoglycoside phosphotransferase | Putative cluster |
| *Nocardioides psychrotolerans* | *Actinobacteria*  *Actinobacteria*  *Propionibacteriales*  *Nocardioidaceae*  *Nocardiodes* | UniRef90_A0A1I3PBM5 | Maltokinase | No cluster detected |
| *Streptosporangium amethystogenes* | *Actinobacteria*  *Actinobacteria*  *Streptosporangiales*  *Streptosporangiaceae*  *Streptosporangium* | UniRef90_UPI0006919BC2 | Hypothetical protein | No cluster detected |
| *Frankia inefficax* | *Actinobacteria*  *Actinobacteria*  *Frankiales*  *Frankiaceae*  *Frankia* | UniRef90_E3JA12 | Aminoglycoside phosphotransferase | Putative cluster |
| *Frankia* sp*.* (strain BMG5.36) | *Actinobacteria*  *Actinobacteria*  *Frankiales*  *Frankiaceae*  *Frankia* | UniRef90_A0A1S1RDJ1 | Aminoglycoside phosphotransferase | Putative cluster |
| *Microbispora* sp*.* (strain GMKU363) | *Actinobacteria*  *Actinobacteria*  *Streptosporangiales*  *Streptosporangiaceae*  *Microbispora* | UniRef90_UPI0006E25C8F | Hypothetical protein | No cluster detected |
| *Nonomuraea* sp*.* (strain SBT364) | *Actinobacteria*  *Actinobacteria*  *Streptosporangiales*  *Streptosporangiaceae*  *Nonomuraea* | UniRef90_UPI00066DC382 | Hypothetical protein | No cluster detected |
| *Frankia* sp*.* (strain DC12) | *Actinobacteria*  *Actinobacteria*  *Frankiales*  *Frankiaceae*  *Frankia* | UniRef90_UPI0005F83CE6 | Aminoglycoside phosphotransferase | Putative cluster |
| *Microbispora* sp*.* (strain ATCC PTA-5024) | *Actinobacteria*  *Actinobacteria*  *Streptosporangiales*  *Streptosporangiaceae*  *Microbispora* | UniRef90_W2EVJ5 | Uncharacterized protein | No cluster detected |
| *Thermoactinospora rubra* | *Actinobacteria*  *Actinobacteria*  *Streptosporangiales*  *Streptosporangiaceae*  *Thermoactinospora* | UniRef90_UPI000A1052BC | Hypothetical protein | No cluster detected |
| *Microtetraspora niveoalba* | *Actinobacteria*  *Actinobacteria*  *Streptosporangiales*  *Streptosporangiaceae*  *Microtetraspora* | UniRef90_UPI000835E86F | Hypothetical protein | No cluster detected |
| *Streptosporangium canum* | *Actinobacteria*  *Actinobacteria*  *Streptosporangiales*  *Streptosporangiaceae*  *Streptosporangium* | UniRef90_A0A1I3I320 | Maltokinase | No cluster detected |
| *Microtetraspora fusca* | *Actinobacteria*  *Actinobacteria*  *Streptosporangiales*  *Streptosporangiaceae*  *Microtetraspora* | UniRef90_UPI000836A88C | Hypothetical protein | No cluster detected |
| *Sinosporangium album* | *Actinobacteria*  *Actinobacteria*  *Streptosporangiales*  *Streptosporangiaceae*  *Sinosporangium* | UniRef90_A0A1G7VBM6 | Maltokinase | No cluster detected |
| *Nonomuraea solani* | *Actinobacteria*  *Actinobacteria*  *Streptosporangiales*  *Streptosporangiaceae*  *Nonomuraea* | UniRef90_A0A1H6B4L3 | Maltokinase | No cluster detected |
| *Nonomuraea jiangxiensis* | *Actinobacteria*  *Actinobacteria*  *Streptosporangiales*  *Streptosporangiaceae*  *Nonomuraea* | UniRef90_A0A1G8VHP3 | Maltokinase | No cluster detected |
| *Nonomuraea wenchangensis* | *Actinobacteria*  *Actinobacteria*  *Streptosporangiales*  *Streptosporangiaceae*  *Nonomuraea* | UniRef90_A0A1I0LT16 | Maltokinase | No cluster detected |
| *Nonomuraea indica* | *Actinobacteria*  *Actinobacteria*  *Streptosporangiales*  *Streptosporangiaceae*  *Nonomuraea* | UniRef90_UPI000C7ACCE8 | Aminoglycoside phosphotransferase | No cluster detected |
| *Planobispora rosea* | *Actinobacteria*  *Actinobacteria*  *Streptosporangiales*  *Streptosporangiaceae*  *Planobispora* | UniRef90_UPI00083A6397 | Hypothetical protein | No cluster detected |
| *Micromonospora narathiwatensis* | *Actinobacteria*  *Actinobacteria*  *Micromonosporales*  *Micromonosporaceae*  *Micromonospora* | UniRef90_A0A1A8ZPS6 | Maltokinase | Type I PKS / NRPS cluster |
| *Nonomuraea candida* | *Actinobacteria*  *Actinobacteria*  *Streptosporangiales*  *Streptosporangiaceae*  *Nonomuraea* | UniRef90_UPI0006948DCE | Hypothetical protein | No cluster detected |
| *Streptosporangium subroseum* | *Actinobacteria*  *Actinobacteria*  *Streptosporangiales*  *Streptosporangiaceae*  *Streptosporangium* | UniRef90_A0A239G8L3 | Maltokinase | No cluster detected |
| *Micromonospora viridifaciens* | *Actinobacteria*  *Actinobacteria*  *Micromonosporales*  *Micromonosporaceae*  *Micromonospora* | UniRef90_A0A1C4XCY9 | Maltokinase | No cluster detected |
| *Millisia brevis* | *Actinobacteria*  *Actinobacteria*  *Corynebacteriales*  *Gordoniaceae*  *Millisia* | UniRef90_UPI000832ABFF | Hypothetical protein | No cluster detected |
| *Nonomuraea* sp*.* (strain ATCC 55076) | *Actinobacteria*  *Actinobacteria*  *Streptosporangiales*  *Streptosporangiaceae*  *Nonomuraea* | UniRef90_A0A1V0A7D7 | Uncharacterized protein | No cluster detected |
| *Herdidospora cretacea* | *Actinobacteria*  *Actinobacteria*  *Streptosporangiales*  *Streptosporangiaceae*  *Herdidospora* | UniRef90_UPI0007744C35 | Hypothetical protein | No cluster detected |
| *Micromonospora* sp*.* (strain CB01531) | *Actinobacteria*  *Actinobacteria*  *Micromonosporales*  *Micromonosporaceae*  *Micromonospora* | UniRef90_A0A1Q4ZS62 | Uncharacterized protein | No cluster detected |
| *Arthrobacter koreensis* | *Actinobacteria*  *Actinobacteria*  *Micrococcales*  *Micrococcaceae*  *Arthrobacter* | UniRef90_UPI000B328D64 | Hypothetical protein | No cluster detected |
| *Actinoplanes subtropicus* | *Actinobacteria*  *Actinobacteria*  *Micromonosporales*  *Micromonosporaceae*  *Actinoplanes* | UniRef90_UPI0006908AF2 | Hypothetical protein | No cluster detected |
| *Arthrobacter luteolus* | *Actinobacteria*  *Actinobacteria*  *Micrococcales*  *Micrococcaceae*  *Arthrobacter* | UniRef90_UPI00082976B6 | Hypothetical protein | No cluster detected |
| *Agromyces cerinus* subsp. *cerinus* | *Actinobacteria*  *Actinobacteria*  *Micrococcales*  *Micrococcaceae*  *Agromyces* | UniRef90_A0A1N6GDG2 | Maltokinase | No cluster detected |
| *Arthrobacter* sp*.* (strain Edens01) | *Actinobacteria*  *Actinobacteria*  *Micrococcales*  *Micrococcaceae*  *Arthrobacter* | UniRef90_A0A0P7GHY6 | Uncharacterized protein | No cluster detected |
| *Agromyces* sp*.* (strain Root81) | *Actinobacteria*  *Actinobacteria*  *Micrococcales*  *Micrococcaceae*  *Agromyces* | UniRef90_A0A0Q8VX32 | Uncharacterized protein | No cluster detected |
| *Agromyces* sp*.* (strain CF514) | *Actinobacteria*  *Actinobacteria*  *Micrococcales*  *Micrococcaceae*  *Agromyces* | UniRef90_A0A1I6INC3 | Maltokinase | No cluster detected |
| *Agromyces* sp*.* (strain Leaf222) | *Actinobacteria*  *Actinobacteria*  *Micrococcales*  *Micrococcaceae*  *Agromyces* | UniRef90_A0A0Q4HCK4 | Uncharacterized protein | No cluster detected |
| *Mumia flava* | *Actinobacteria*  *Actinobacteria*  *Propionibacteriales*  *Nocardioidaceae*  *Mumia* | UniRef90_A0A2M9B6V7 | Maltokinase | No cluster detected |
| *Sphaerisporangium album* | *Actinobacteria*  *Actinobacteria*  *Streptosporangiales*  *Streptosporangiaceae*  *Streptosporangium* | UPI000DE91F25 (UniParc) | Aminoglycoside phosphotransferase | No cluster detected |
| *Jishengella* sp*.* (strain NA12) | *Actinobacteria*  *Actinobacteria*  *Micromonosporales*  *Micromonosporaceae*  *Micromonospora* | UniRef90_A0A2W2DCK5 | Aminoglycoside phosphotransferase | No cluster detected |
| *Homoserinimonas* sp. (strain OAct 916) | *Actinobacteria*  *Actinobacteria*  *Micrococcales*  *Microbacteruaceae*  *Homoserinimonas* | UniRef90_UPI000DBEA3B7 | Hypothetical protein | No cluster detected |
| *Actinoplanes lutulentus* | *Actinobacteria*  *Actinobacteria*  *Micromonosporales*  *Micromonosporaceae*  *Actinoplanes* | UniRef90_UPI000DB94A95 | Hypothetical protein | No cluster detected |
| *Nonomuraea fuscirosea* | *Actinobacteria*  *Actinobacteria*  *Streptosporangiales*  *Streptosporangiaceae*  *Nonomuraea* | UniRef90_A0A2T0MNG9 | Maltokinase | No cluster detected |
| *Mycobacterium* sp. (strain ITM-2016-00316) | *Actinobacteria*  *Actinobacteria*  *Corynebacteriales*  *Mycobacteriaceae*  *Mycobacterium* | UniRef90_A0A2S8LAU3 | Aminoglycoside phosphotransferase | No cluster detected |
