# Supplementary material for: Molecular Fingerprints for a Novel Enzyme Family in Actinobacteria with Glucosamine Kinase Activity
Source: mBio. 2019 May 14;10(3):e00239-19. doi: 10.1128/mBio.00239-19 (PMC6520443; doi:10.1128/mBio.00239-19)
Supplement: TEXT S1 [file mBio.00239-19-s0001.docx]

Supplemental Methods for

Molecular fingerprints for a novel enzyme family in *Actinobacteria* with glucosamine kinase activity

José A. Manso,^a,b^ Daniela Nunes-Costa,^c,d^ Sandra Macedo-Ribeiro,^a,b^ Nuno Empadinhas,^c,e^#

Pedro J. B. Pereira,^a,b^#

^a^IBMC - Instituto de Biologia Molecular e Celular, Universidade do Porto, Porto, Portugal.

^b^i3S - Instituto de Investigação e Inovação em Saúde, Universidade do Porto, Porto, Portugal.

^c^CNC - Center for Neuroscience and Cell Biology, University of Coimbra, Coimbra, Portugal.

^d^PhD Program in Experimental Biology and Biomedicine (PDBEB), University of Coimbra, Coimbra Portugal.

^e^IIIUC-Institute for Interdisciplinary Research, University of Coimbra, Coimbra Portugal.

**Cloning and site-directed mutagenesis**

Chromosomal DNA from *S. jiangxiensis* and from *M. smegmatis* was isolated with the Microbial gDNA Isolation kit (NZYTech). The DNA sequences encoding *S. jiangxiensis* kinase (UniProtKB entry A0A1H7TQR5) and *M. smegmatis* kinase (UniProtKB entry A0A0D6IZ29) were amplified by PCR using KOD Hot-Start DNA polymerase (Novagen) with forward primers 5’-ACATTTCTGCATATGACCCCGAACTGGT and 5’-TAATATCATATGATCGAGCTCGACC and reverse primers 5’-GATCAAGCTTGTCCTTCAGTCTTTCCG and 5’- TATAAGCTTCGTGCTTGTCCC, respectively (restriction sites underlined). Amplification products were cloned into the NdeI and HindIII sites of pET-30a(+) bacterial expression vector (Novagen), in frame with the vector-encoded C-terminal hexahistidine tag. Single point mutations were introduced by site-directed mutagenesis using the QuikChange method (Stratagene).

**Determination of kinetic parameters**

Kinase activity assays were performed with the ADP-Glo^TM^ kinase Assay Kit (Promega) (H. Zegzouti, M. Zdanovskaia, K. Hsiao, and S. A. Goueli, Assay Drug Dev Technol, 7:560–572, 2009, doi:10.1089/adt.2009.0222). The reaction mix (containing enzyme, ATP and sugar of interest at different concentrations) was prepared in 100 m*M* Tris pH 7.5, 20 m*M* MgCl_2_, 0.1 mg mL^-1^ BSA. The mix (5 μL) was incubated in an opaque white 384-well assay plate (Corning) at room temperature (RT) for 5 min, followed by addition of the ADP-Glo^TM^ reagent (5 μL per well) and incubation for 1 h at RT. Finally, 10 μL of kinase detection reagent were added to each well and the plate was again incubated for 1 h at RT. The luminescence was then recorded using a Synergy 2 plate reader (BioTek) and the data were analyzed with SigmaPlot (Systat Sofware). ATP to ADP standard curves were prepared in the 0.01 μ*M* to 200 μ*M* concentration range.

**Crystallization**

All SjGlcNK crystals were obtained by sitting drop vapor diffusion at 20 °C. Crystals belonging to crystal form A grew from drops composed of 1 μL protein solution (45 mg mL^-1^ in 20 m*M* BTP pH 7.4, 50 m*M* NaCl) and 2 μL precipitant (100 m*M* Bis-Tris pH 6.1, 15% (wt/vol) PEG 3350, 0.2 *M* MgCl_2_). Prior to data collection the crystals were transferred to a 1:1 mixture of mineral oil and Parabar 10312 (Hampton Research) and flashed-cooled in liquid N_2_. Crystals of SjGlcNK belonging to crystal form B were obtained from drops composed of 1 μL protein solution (45 mg mL^-1^ in 20 m*M* BTP pH 7.4, 50 m*M* NaCl, pre-incubated for 2 h at 4 °C with 10 m*M* GlcN in the same buffer) and 2 μL crystallization solution (100 m*M* Bis-Tris pH 5.7, 16.5% (wt/vol) PEG 3350, 0.15 *M* MgCl_2_). Prior to data collection the crystals were cryoprotected in crystallization solution supplemented with 20% (vol/vol) glycerol and flashed-cooled in liquid N_2_. Crystals of SjGlcNK in complex with ADP, Pi, and GlcN were obtained from drops composed of 1 μL protein solution (45 mg mL^-1^ in 20 m*M* BTP pH 7.4, 50 m*M* NaCl, pre-incubated for 1 h at 4 °C with 0.2 *M* GlcN, 10 m*M* ATP in the same buffer) and 2 μL precipitant (100 m*M* Bis-Tris pH 6.1, 16% (wt/vol) PEG 3350, 0.15 *M* MgCl_2_). Prior to data collection the crystals were transferred to crystallization solution supplemented with 20% (wt/vol) PEG400 and flashed-cooled in liquid N_2_.

**Data collection and processing**

All diffraction data were collected at 100 K. Data from crystal form A (doi:10.15785/SBGRID/614) were collected on the BL13-XALOC beamline (J. Juanhuix, F. Gil-Ortiz, G. Cuní, C. Colldelram, J. Nicolás, J. Lidón, E.. Boter, C. Ruget, S. Ferrer, and J. Benach, J Synchrotron Radiat, 21:679-689, 2014, doi:10.1107/S160057751400825X) of the ALBA-CELLS synchrotron (Cerdanyola del Vallès, Spain), and data from crystal form B (doi:10.15785/SBGRID/614) and from the complex with ADP, Pi, and GlcN (doi:10.15785/SBGRID/616) on the ID30A-3 beamline (P. Theveneau, R. Baker, R. Barrett, A. Beteva, M. W. Bowler, P. Carpentier, H. Caserotto, D. de Sanctis, F. Dobias, D. Flot, M. Guijarro, T. Giraud, M. Lentini, G. A. Leonard, M. Mattenet, A. A. McCarthy, S. M. McSweeney, C. Morawe, M. Nanao, D. Nurizzo, S. Ohlsson, P. Pernot, A. N. Popov, A. Round, A. Royant, W. Schmid, A. Snigirev, J. Surr, and C. Mueller-Dieckmann, J Phys: Conf Ser, 425:012001, 2013, doi:10.1088/1742-6596/425/1/012001) of the European Synchrotron Radiation Facility (Grenoble, France). Diffraction data were processed with the programs XDS (W. Kabsch, XDS. Acta Crystallogr D Biol Crystallogr, 66:125-132, 2010, doi:10.1107/S0907444909047337), Pointless (P. Evans, Acta Crystallogr D Biol Crystallogr, 62:72-82, 2006, doi:10.1107/S0907444905036693), and Aimless (P. R. Evans, and G. N. Murshudov, Acta Crystallogr D Biol Crystallogr, 69:1204-1214, 2013, doi:10.1107/S0907444913000061) as implemented in the autoPROC pipeline (C. Vonrhein, C. Flensburg, P. Keller, A. Sharff, O. Smart, W. Paciorek, T. Womack, and G. Bricogne, Acta Crystallogr D Biol Crystallogr, 67:293-302, 2011, doi:10.1107/S0907444911007773). Crystals belong to the monoclinic space group *P*2_1_ (S1 Table) and those of crystal form A and of the complex with ADP, Pi, and GlcN contain two monomers of SjGlcNK in the AU (46% solvent content), while those of crystal form B contain four monomers of SjGlcNK in the AU (51% solvent content).

**Structure solution and refinement**

The structure of SjGlcNK was solved by molecular replacement with Phaser (A. J. McCoy, R. W. Grosse-Kunstleve, P. D. Adams, M. D. Winn, L. C. Storoni and R. J. Read, J Appl Crystallogr, 40:658-674, 2007, doi:10.1107/S0021889807021206) as implemented in the MrBUMP pipeline (R. M. Keegan and M. D. Winn, Acta Crystallogr D Biol Crystallogr, 64:119-124, 2008, doi:10.1107/S0907444907037195) from the CCP4 suite (M. D. Winn, C. C. Ballard, K. D. Cowtan, E. J. Dodson, P. Emsley, P. R. Evans, R. M. Keegan, E. B. Krissinel, A. G. W. Leslie, A. McCoy, S. J. McNicholas, G. N. Murshudov, N. S. Pannu, E. A. Potterton, H. R. Powell, R. J. Read, A. Vagin, and K. S. Wilson, Acta Crystallogr D Biol Crystallogr, 67:235-242, 2011, doi:10.1107/S0907444910045749) using data from crystal form A. A marginal solution was found using an ensemble composed by the models of the mycobacterial maltokinases MvMak (PDB entry 4U94) (J. Fraga, A. Maranha, V. Mendes, P. J. B. Pereira, N. Empadinhas, and S. Macedo-Ribeiro, Sci Rep 5:8026, 2015, doi:10.1038/srep08026) and MtMak (PDB entry 4O7O) (J. Li, X. Guan, N. Shaw, W. Chen, Y. Dong, X. Xu, X. Li, and Z. Raoa, Sci Rep, 4:6418, 2014, doi:10.1038/srep06418), but after some rounds of automatic restrained refinement with REFMAC (G. N. Murshudov, P. Skubák, A. A. Lebedev, N. S. Pannu, R. A. Steiner, R. A. Nicholls, M. D. Winn, F. Long, and A. A. Vagin, Acta Crystallogr D Biol Crystallogr. 67:355-367, 2011, doi:10.1107/S0907444911001314) the *R*_free_ was stuck at 0.52. This model was then subjected to smooth deformation with the morph module of Phenix (T. C. Terwilliger, R. J. Read, P. D. Adams, A. T. Brunger, P. V. Afonine and L-W. Hung, Acta Crystallogr D Biol Crystallogr, 69:2244-2250, 2013, doi:10.1107/S0907444913017770) and after 25 morphing cycles (6 Å radius of morphing) there was a substantial improvement in the quality of the electron density maps, accompanied by vastly improved statistics (*R*_work_ = 0.43 and *R*_free_ = 0.47). This initial model was refined with Phenix (P. D. Adams, P. V. Afonine, G. Bunkóczi, V. B. Chen, I. W. Davis, N. Echols, J. J. Headd, L.-W. Hung, G. J. Kapral, R. W. Grosse-Kunstleve, A. J. McCoy, N. W. Moriarty, R. Oeffner, R. J. Read, D. C. Richardson, J. S. Richardson, T. C. Terwilliger, and P. H. Zwart, Acta Crystallogr D Biol Crystallogr, 66:213-221, 2010, doi:10.1107/S0907444909052925), alternating with manual model building with Coot (P. Emsley, B. Lohkamp, W. G. Scott, and K. Cowtan, Acta Crystallogr D Biol Crystallogr, 66:486-501, 2010, doi:10.1107/S0907444910007493). At this stage, only the C-lobe (residues 181-438) could be modeled for the two molecules in the AU. The remaining of molecule A (180 residues) was then built with ARP/wARP (G. Langer, S. X. Cohen, V. S. Lamzin, and A. Perrakis, Nat Protoc, 3:1171-1179, 2008, doi:10.1038/nprot.2008.91). Finally, the missing segment of molecule B was placed using Phaser and residues 4-180 of molecule A as search model. It then became evident that the two molecules of SjGlcNK in the AU display different conformational states, explaining the difficulties experienced during the phasing stage. The model was completed with alternating cycles of refinement with Phenix (P. D. Adams, P. V. Afonine, G. Bunkóczi, V. B. Chen, I. W. Davis, N. Echols, J. J. Headd, L.-W. Hung, G. J. Kapral, R. W. Grosse-Kunstleve, A. J. McCoy, N. W. Moriarty, R. Oeffner, R. J. Read, D. C. Richardson, J. S. Richardson, T. C. Terwilliger, and P. H. Zwart, Acta Crystallogr D Biol Crystallogr, 66:213-221, 2010, doi:10.1107/S0907444909052925) and manual model building with Coot (P. Emsley, B. Lohkamp, W. G. Scott, and K. Cowtan, Acta Crystallogr D Biol Crystallogr, 66:486-501, 2010, doi:10.1107/S0907444910007493). The final refined model includes residues 1-89, 95-118, and 122-436 of molecule A, and residues 1-19, 33-66, 69-88, 97-115, 123-174, and 178-441 of molecule B, with 97.7% of the main-chain torsion angles in the favored regions of the Ramachandran plot.

The structure of SjGlcNK crystal form B was solved by molecular replacement with Phaser (A. J. McCoy, R. W. Grosse-Kunstleve, P. D. Adams, M. D. Winn, L. C. Storoni and R. J. Read, J Appl Crystallogr, 40:658-674, 2007, doi:10.1107/S0021889807021206) using two fragments (residues 1-188 and 189-433) of molecule A from crystal form A as search models. The resulting solution was refined with Phenix (P. D. Adams, P. V. Afonine, G. Bunkóczi, V. B. Chen, I. W. Davis, N. Echols, J. J. Headd, L.-W. Hung, G. J. Kapral, R. W. Grosse-Kunstleve, A. J. McCoy, N. W. Moriarty, R. Oeffner, R. J. Read, D. C. Richardson, J. S. Richardson, T. C. Terwilliger, and P. H. Zwart, Acta Crystallogr D Biol Crystallogr, 66:213-221, 2010, doi:10.1107/S0907444909052925), alternating with cycles of manual model building with Coot (P. Emsley, B. Lohkamp, W. G. Scott, and K. Cowtan, Acta Crystallogr D Biol Crystallogr, 66:486-501, 2010, doi:10.1107/S0907444910007493). The final refined model (96.5% of the main-chain torsion angles in the favored regions of the Ramachandran plot) comprises residues 4-20, 28-65, 72-89, 93-115, and 121-436 for molecule A, 3-11, 14-19, 35-63, 70-113, and 121-435 for molecule B, 4-20, 27-65, 70-89, 95-117, 122-173 and 177-433 for molecule C, and 3-23, 29-89, 96-117, and 123-434 for molecule D.

Molecular replacement with Phaser (A. J. McCoy, R. W. Grosse-Kunstleve, P. D. Adams, M. D. Winn, L. C. Storoni and R. J. Read, J Appl Crystallogr, 40:658-674, 2007, doi:10.1107/S0021889807021206) using molecules A and B of SjGlcNK crystal form A as search models gave a straightforward solution for the complex with GlcN, ADP and Pi. The model was completed with alternating cycles of refinement with Phenix (P. D. Adams, P. V. Afonine, G. Bunkóczi, V. B. Chen, I. W. Davis, N. Echols, J. J. Headd, L.-W. Hung, G. J. Kapral, R. W. Grosse-Kunstleve, A. J. McCoy, N. W. Moriarty, R. Oeffner, R. J. Read, D. C. Richardson, J. S. Richardson, T. C. Terwilliger, and P. H. Zwart, Acta Crystallogr D Biol Crystallogr, 66:213-221, 2010, doi:10.1107/S0907444909052925) and manual model building with Coot (P. Emsley, B. Lohkamp, W. G. Scott, and K. Cowtan, Acta Crystallogr D Biol Crystallogr, 66:486-501, 2010, doi:10.1107/S0907444910007493). The final refined model (97.4% of the main-chain torsion angles in the favored regions of the Ramachandran plot) comprises residues 1-19, 26-89, 94-117 and 121-433 for molecule A and 4-8, 14-20, 33-63, 72-90, 96-104, 111-116, 121-141 and 143-441 for molecule B. Detailed refinement statistics are given in S1 Table. All crystallographic software was supported by SBGrid (A. Morin, B. Eisenbraun, J. Key, P. C. Sanschagrin, M. A. Timony, M. Ottaviano, and P. Sliz, eLife, 2:e01456, 2013, doi:10.7554/eLife.01456).

**SAXS measurements and analysis**

SAXS data were collected at BioSAXS beamline BM29 (P. Pernot, A. Round, R. Barrett, A. De Maria Antolinos, A. Gobbo, E. Gordon, J. Huet, J. Kieffer, M. Lentini, M. Mattenet, C. Morawe, C. Mueller-Dieckmann, S. Ohlsson, W. Schmid, J. Surr, P. Theveneau, L. Zerrad, and S. McSweeney, J Synchrotron Radiat, 20:660-664, 2013, doi:10.1107/S0909049513010431) of the European Synchrotron Radiation Facility (Grenoble, France). The SjGlcNK samples were equilibrated in 20 m*M* Tris-HCl pH 8.0, 150 m*M* NaCl, 10 m*M* MgCl_2_, 5 m*M* DTT by size exclusion chromatography (see *Protein expression and purification*, above, for details), concentrated by ultrafiltration on a 10 kDa molecular weight cutoff centrifugal device (Millipore), and centrifuged at 21,500 *g* and 4 °C for 30 min to remove possible aggregates. Mixtures of SjGlcNK (10 mg mL^-1^) with 200 m*M* GlcN, 50 m*M* glucose or 1 m*M* ATP, and the different sugar/nucleotide combinations at these concentrations were prepared and immediately flash frozen in liquid nitrogen. Matching buffers were prepared by adding the adequate amount of ligand(s) to SjGlcNK protein buffer. Prior to data collection, thawed samples were centrifuged at 21,500 *g* and 4 °C for 10 min. All samples and their corresponding buffers were measured consecutively in standard "batch" mode using the automated sample changer, which ensures continuous flow. In order to evaluate the magnitude of interparticle effects, the samples were measured at four concentrations, in the range of 1.25-10.0 mg mL^-1^ or 0.625-5.0 mg mL^-1^, obtained by 2-fold serial dilution of the most concentrated sample. The images (frames) were collected over a scattering vector from 0.0035 to 0.5 Å^-1^ (*q* = (4π sin*θ*)/*λ*, where 2*θ* is the scattering angle). Data were processed and analyzed with the *ATSAS* 2.8 package (D. Franke, M. V. Petoukhov, P. V. Konarev, A. Panjkovich, A. Tuukkanen, H. D. T. Mertens, A. G. Kikhney, N. R. Hajizadeh, J. M. Franklin, C. M. Jeffries, and D. I. Svergun, J Appl Crystallogr, 50:1212-1225, 2017, doi:10.1107/S1600576717007786). Extrapolation from multiple scattering curves at different concentrations to an infinite dilution and Guinier analysis were done with *PRIMUS*/*qt* (M. V. Petoukhov, D. Franke, A. V. Shkumatov, G. Tria, A. G. Kikhney, M. Gajda, C. Gorba, H. D. T. Mertens, P. V. Konarev, and D. I. Svergun, J Appl Crystallogr, 45:342-350, 2012, doi:10.1107/S0021889812007662). The *R*_g_ remained constant for all concentrations, within the experimental error. Slight interparticle effects were observed for the two most concentrated samples of apo-SjGlcNK and in presence of GlcN. Hence, to minimize any interparticle effect contribution, the corresponding scattering curves were not used in the extrapolation to infinite dilution. The pair-distance distribution function, *P*(*r*), was calculated with *GNOM* (D. I. Svergun, J Appl Crystallogr, 25:495-503, 1992, doi:10.1107/S0021889892001663), scattering profiles of atomic structures with *CRYSOL* (D. Svergun, C. Barberato, and M. H J. Koch, J Appl Crystallogr, 28:768-773, 1995, doi:10.1107/S0021889895007047), and the volume fractions for the open (I) and closed (VI) conformations of SjGlcNK with *OLIGOMER* (P. V. Konarev, V. V. Volkov, A. V. Sokolova, M. H. J. Koch, and D. I. Svergun. J Appl Crystallogr, 36:1277-1282, 2003, doi:10.1107/S0021889803012779).

**Structure and sequence analysis**

Subdomain motions were analyzed with DynDom (S. Hayward and H. J. Berendsen, Proteins, 30:144-154, 1998, doi:10.1002/(SICI)1097-0134(19980201)30:2<144::AID-PROT4>3.0.CO;2-N) and evolutionary conservation scores were calculated with Consurf (H. Ashkenazy, S. Abadi, E. Martz, O. Chay, I. Mayrose, T. Pupko, and N. Ben-Tal, Nucleic Acids Res, 44:W344-W350, 2016, doi:10.1093/nar/gkw408). PC analysis was carried out on the collection of experimental structures using the GROMACS utilities (S. Pronk, S. Páll, R. Schulz, P. Larsson, P. Bjelkmar, R. Apostolov, M. R. Shirts, J. C. Smith, P. M. Kasson, D. van der Spoel, B. Hess, and E. Lindahl, Bioinformatics. 29:845-854, 2013, doi:10.1093/bioinformatics/btt055).
